# Targeted modulation of intestinal barrier and mucosal immune-related microbiota attenuates IgA nephropathy progression

**DOI:** 10.1080/19490976.2025.2458184

**Published:** 2025-01-28

**Authors:** Ran Zhang, Yuyan Tang, Xiangru Feng, Xiaoxuan Lu, Mengyao Zhao, Jiayang Jin, Xiaoguo Ji, Haidong He, Liming Zhao

**Affiliations:** a State Key Laboratory of Bioreactor Engineering, East China University of Science and Technology, Shanghai, China; b Shanghai Frontiers Science Center of Optogenetic Techniques for Cell Metabolism, School of Pharmacy, East China University of Science and Technology, Shanghai, China; c Department of Nephrology, Minhang Hospital, Fudan University, Shanghai, China; d Shanghai Collaborative Innovation Center for Biomanufacturing Technology (SCICBT), Shanghai, China

**Keywords:** IgA nephropathy, gut microbiota, chitooligosaccharides, kidney-gut crosstalk, human microbiota-associated mice

## Abstract

IgA nephropathy (IgAN) is related to the balance of gut microbiota. However, it is unclear whether changes in the gut microbiota can cause IgAN or attenuate its progression. This study employed IgAN and human microbiota-associated (HMA)-IgAN models to investigate the impact of IgAN on gut microbiota alteration and the mechanisms by which gut microbiota might trigger IgAN. Furthermore, this study examined the effects of chitooligosaccharides (COS) and COS formulation (COSF) with microbiota-targeting function on enhancing intestinal barrier and renal functions. These results revealed that IgAN led to a reduction in α-diversity and structural alterations in the gut microbiota, characterized by an increase in *Shigella sonnei*, *Streptococcus danieliae*, *Desulfovibrio fairfieldensis*, and a decrease in *Bifidobacterium pseudolongum* and *Clostridium leptum*. There was also an imbalance in intestinal B-cell immunity and a decrease in the level of tight junction proteins (ZO-1 and Occludin). Intestinal barrier and mucosal immune-related microbiota (*Clostridium leptum, unclassified Lachnospiraceae NK4Al36 group, unclassified Clostridia vadinBB60 group, unclassified Oscillospiraceae*, and *unclassified Roseburia*) were enriched through targeted modulation with COS/COSF, enhancing intestinal ZO-1 expression and reducing APRIL/BAFF overexpression, thereby reducing renal damage in IgAN. In conclusion, this study clarified the kidney-gut crosstalk between gut microbiota and IgAN, providing scientific evidence for developing microbiota-targeted food interventions to improve IgAN outcomes.

## HIGHLIGHTS

1. IgAN disrupted specific microbial community, affecting barrier and immunity.
2. The specific microbial community can affect ZO-1, Occludin, and B-cell immunity, contributing to IgAN disease.
3. Renal injury in IgAN was repaired by targeting the intestinal barrier and mucosal immune-related microbiota.
4. Targeted modulation of gut microbiota alleviated intestinal B cell immune imbalance by inhibiting APRIL/BAFF overproduction.

## GRAPHICAL ABSTRACT

**Figure:**
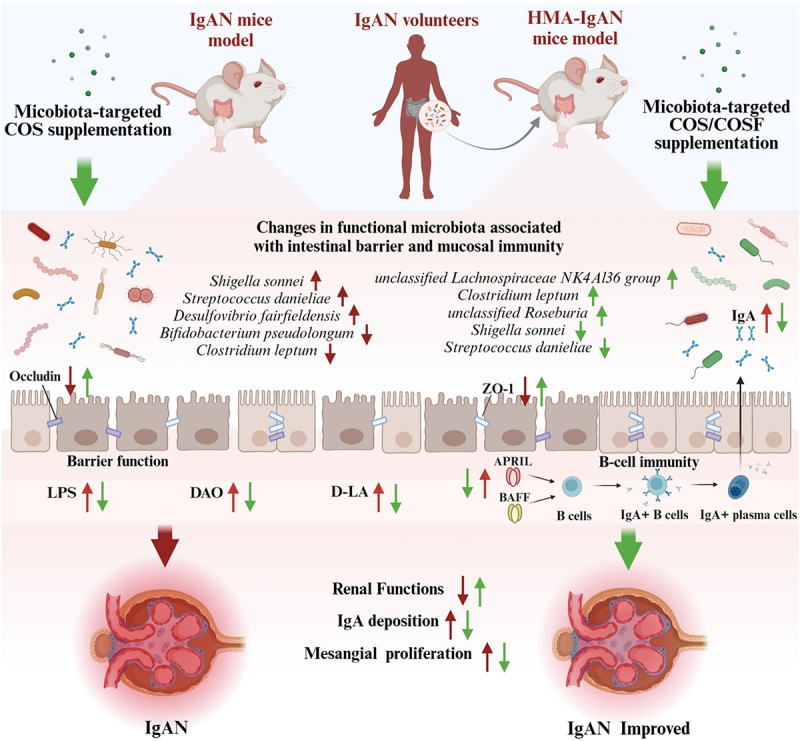

## Introduction

IgA nephropathy (IgAN) is the most common immune-mediated primary glomerulonephritis worldwide, with an incidence of at least 2.5 per 100,000 adults annually. It carries a significant lifetime risk of progressing to end-stage renal disease (ESRD),^1,2^ with approximately 20% to 40% of IgAN patients progressing to ESRD within 10 to 20 years.^3^ There is an urgent need for identifying effective therapeutic targets and novel intervention strategies.

As a bridge between the environment and humans, the gut microbiota plays a fundamental role in the onset and progression of kidney diseases.^4^ Previous studies have shown that the gut microbiota composition of IgAN patients differed from that of healthy controls, with significant increases in *Bacteroides*, *Escherichia*, and *Streptococcus* and decreases in *Lactobacillus*, *Bifidobacterium*, *Clostridium*, *Lachnospira*, and *Prevotella*.^5–7^ Moreover, there was a significant decrease in the levels of fatty acids (particularly unsaturated fatty acids) in the intestinal metabolites and short-chain fatty acids (SCFAs) in the fecal samples of IgAN patients.^8^ Dysbiosis of the gut microbiome also affected host immunity, specifically the production and O-glycosylation of IgA1, which mediated the onset of IgAN.^9,10^ Studies have found that germ-free mice exhibited reduced IgA production, which increased when the abundance of *Bacteroides ovatus* and *Proteobacteria spp*. was simultaneously elevated.^11,12^

The gut microbiota can also mediate immunoregulation and signal transduction through its metabolic products. For instance, reducing polyunsaturated fatty acids (PUFA) and SCFAs in the serum of IgAN patients led to intestinal inflammation and immune hyperactivation.^13^ Uremic toxins generated by the gut microbiota can also stimulate the overproduction of IgA. IgA has been reported to fine-tune the balance between immunity and gut microbiota.^14^ Therefore, the imbalance in gut homeostasis can affect the progression of IgAN. However, the mechanisms through which gut microbiota affect IgAN are still undefined. It is also unclear whether regulating gut microbiota can improve IgAN.

Prebiotics and other functional natural products have significant effects on the composition and function of the gut microbiota. It has been reported that supplementation with inulin-type fructans modified the gut microbiota of diabetic nephropathy animal models and lead to increased levels of acetates, which had a positive impact on diabetic nephropathy.^15^ Peony bark polysaccharides can repair the intestinal barrier function in diabetic nephropathy rats, increase the production of SCFAs, and enrich *Lactobacillus* and *unclassified Muribaculaceae* in a dose- and time-dependent manner.^16^ Studies have shown that dietary fiber supplementation (mixtures of galactomannan, resistant dextrin and fructooligosaccharides) increased the abundance of *Bifidobacterium adolescentis*, *Lactobacillus*, and *Lactobacillaceae*, and serum SCFAs, thereby facilitating renal anemia in patients with ESRD.^17^ The natural product isorhamnetin inhibited the levels of uremic toxins (indoles and indoxyl sulfate) produced by the gut microbiota by regulating the electron transport chain of gut microbiota.^18^ Additionally, marine functional oligosaccharides, known as COS, exhibited prebiotic properties and had a beneficial regulatory effect on the gut microbiota,^19^ intestinal barrier,^20^ mucosal immunity,^21^ and inflammation.^22^ Functional oligosaccharides with probiotic effects can remodel the gut microbiota and revise the metabolomic profile, which may effectively improve IgAN. However, the microbiota targeted for IgAN remains unidentified, and the mechanisms by which microbiota-targeted regulation affects IgAN are not well understood.

This study postulated that the disruption of intestinal barrier and mucosal immunity-related microbiota was related to the pathogenesis of IgAN and that targeting the microbiota with functional oligosaccharides could enhance intestinal barrier integrity and modulate mucosal immunity, potentially decelerating IgAN progression. This study investigated the effects of specific microbial community changes on IgAN by establishing IgAN animal models and human microbiota-associated (HMA)-IgAN animal models. The study verified the bidirectional relationship between the occurrence and development of IgAN and gut microbiota dysbiosis. Moreover, COS and COS-based formulations (COSF), which were microbiota-targeted functional oligosaccharides, could effectively improve IgAN by regulating the intestinal barrier and mucosal immunity-related microbiota.

## Results

### IgAN-induced renal injury and decreased renal function

Renal IgA immunofluorescence staining was analyzed to determine whether the IgAN model had been successfully established. The IgAN group showed a significant increase in glomerular IgA (*p* < 0.001), C3 (*p* < 0.01), and IgG (*p* < 0.001) deposition compared to the control group (Figure 2a–d). PAS staining revealed glomerular mesangial cell proliferation, increased mesangial matrix, and thickened basement membrane in the IgAN group (Figure 2e). Evaluation of renal function indicators showed a significant decline in renal function in the IgAN group, possibly related to kidney structural damage. Specifically, compared to the control group, mice in the IgAN group exhibited significant increases in blood urea nitrogen (BUN) (*p* < 0.001), uric acid (UA) (*p* < 0.01), serum creatinine (Scr) (*p* < 0.01), blood albumin (BAL) (*p* < 0.05), and urine protein/creatinine ratio (ACR) (*p* < 0.001) levels (Figure 2f–j). These aforementioned results confirmed the successful establishment of the IgAN mice model. The increased BLA levels might be related to liver damage and systemic inflammation caused by the modeling agents carbon tetrachloride (CCl_4_) and lipopolysaccharide (LPS).

**Figure 1.**
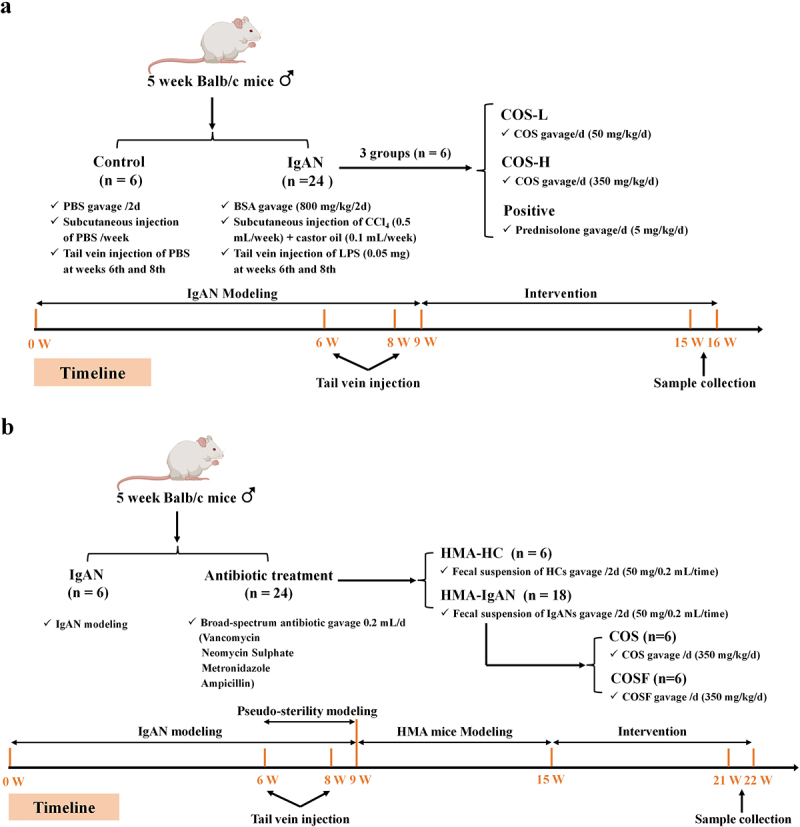
Animal experiment design. a construction of the IgAN model and COS intervention experimental for the IgAN model. b construction of human-microbiota-associated (HMA) animals and the COS/COSF intervention experimental scheme for HMA-IgAN models. HMA-animal models were able to mimic the intestinal state of humans and were used for studies closer to the clinic.

**Figure 2.**
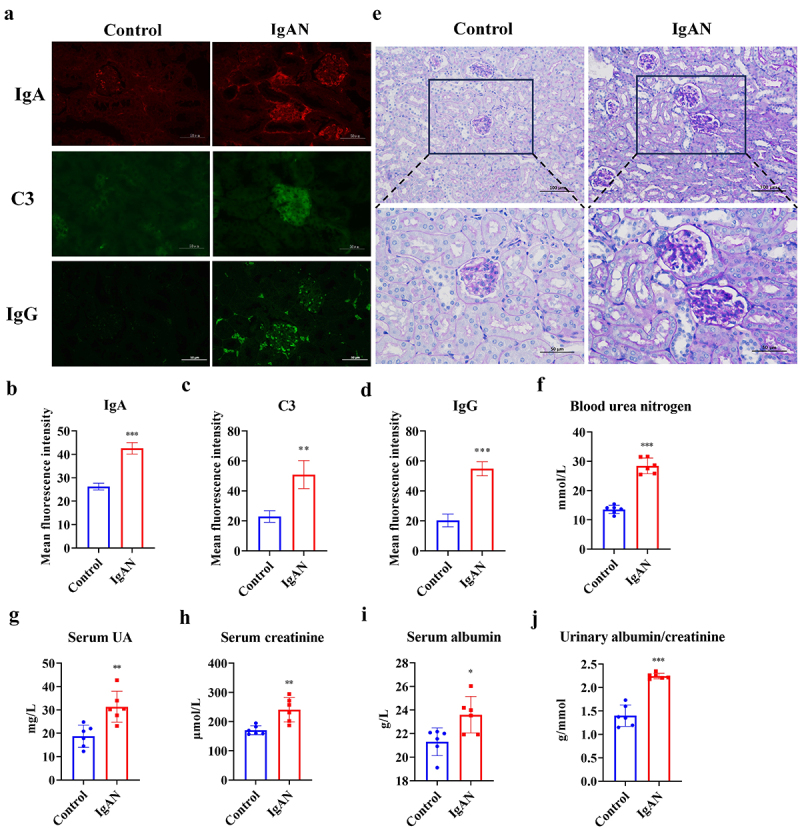
Identification of the IgAN model. a–d Glomerular IgA, C3, and IgG immunofluorescence and quantitative analysis of IgAN vs. Control groups (scale = 50 μm, *n* = 3). e PAS staining of kidneys in IgAN vs. Control groups (scales = 100 μm and 50 μm, respectively, *n* = 3). f–j Assessment of renal function indices in IgAN vs. Control groups (*n* = 6). *Represented *p* < 0.05, **Represented *p* < 0.01, and ***Represented *p* < 0.001.

### IgAN-induced disordered structure and diversity imbalance of gut microbiota

**Figure 3.**
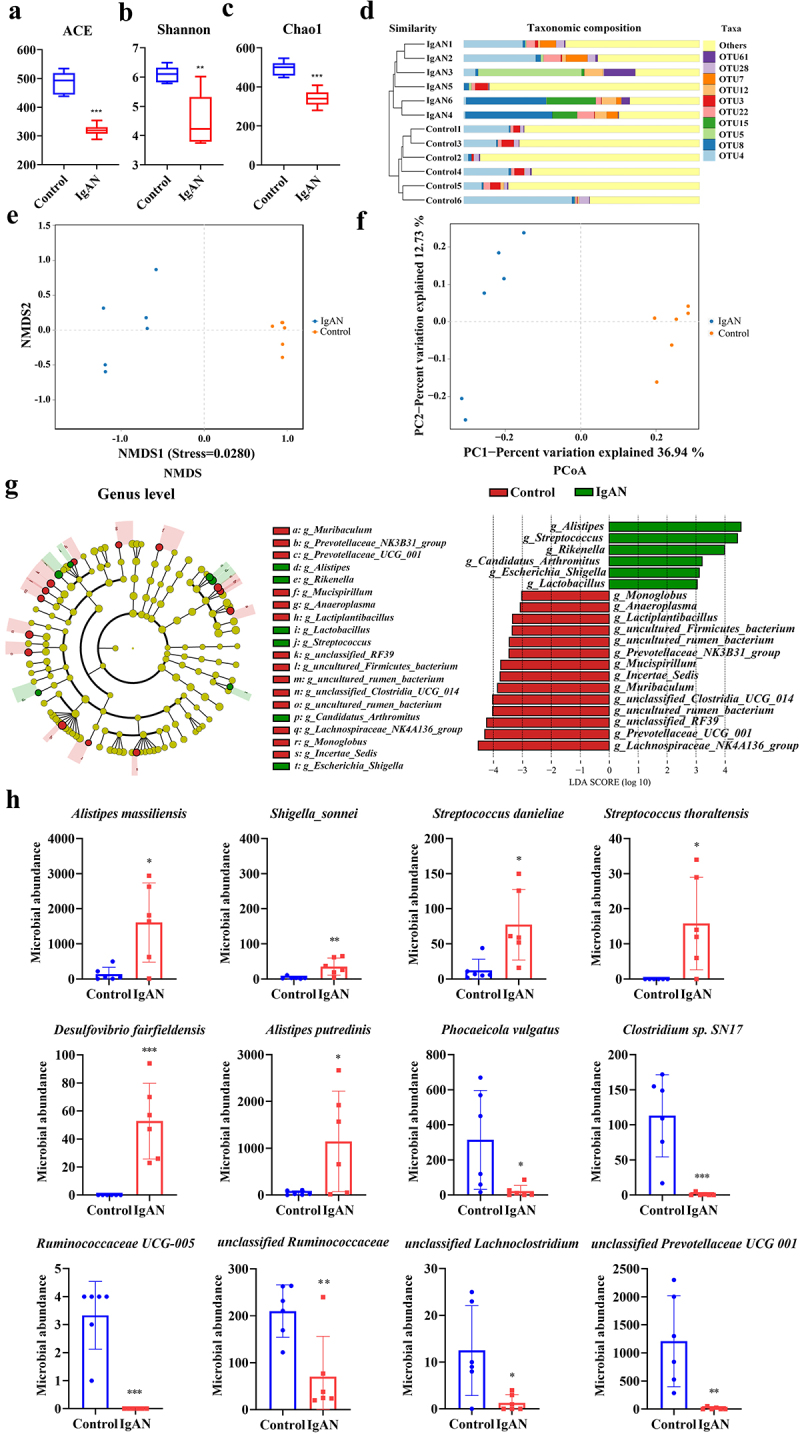
IgAN-induced gut microbiota dysbiosis and decreased diversity (IgAN vs. Control group) (*n* = 6). a–c α-diversity indices of gut microbiota. d UPGMA analysis of samples between the two groups. e NMDS analysis of gut microbiota at the OTU level. f PCoA analysis of gut microbiota at the OTU level. g LEfSe analysis at the genus level and the score histograms. h comparison of species-level differential taxa between the two groups. * represented *p* < 0.05, ** represented *p* < 0.01, and *** represented *p* < 0.001.

Gut microbiota diversity underpins the stability and bioactivity of the gut ecosystem. Patients with IgAN showed significantly reduced gut microbiota diversity and a significantly altered structure compared to healthy controls (Figure S1). We also found that the serum and fecal metabolomes of IgAN patients were significantly altered, in support of the changes in their microbiota (Figure S2-3). Through the construction of an IgAN animal model, we found that the IgAN group exhibited a significant decrease in Ace (*p* < 0.001), Shannon (*p* < 0.01), and Chao1 (*p* < 0.001) indices compared to the control group (Figure 3a–c). Further, UPGMA tree analysis, Non-metric multidimensional scaling (NMDS), and Principal coordinates analysis (PCoA) were used for similarity clustering analysis (Figure 3d–f). These results showed that the IgAN group exhibited distinct microbial clustering from the control group, with clear partitioning between the two groups, indicating that IgAN induced a shift in the microbial communities. To identify the effective characteristics of the gut microbiota of IgAN, the LEfSe (Line Discriminant Analysis (LDA) Effect Size) analysis was conducted (Figure 3g). The IgAN group exhibited significant increases at the genus level in *Alistipes*, *Streptococcus*, *Rikenella*, *Candidatus Arthromitus*, *Escherichia Shigella*, and *Lactobacillus*. At the species level, increases in *Alistipes massiliensis* (*p* < 0.05), *Shigella sonnei* (*p* < 0.01), *Streptococcus danieliae* (*p* < 0.05), *Streptococcus thoraltensis* (*p* < 0.05), *Desulfovibrio fairfieldensis* (*p* < 0.001), and *Alistipes putredinis* (*p* < 0.05) were observed in the IgAN group. In contrast, the control group exhibited higher abundances of *Phocaeieola vulgatus* (*p* < 0.05), *Clostridium sp. SN17* (*p* < 0.001), *Ruminococcacene UCG-005* (*p* < 0.001), *unclassified Ruminococcaceae* (*p* < 0.01), *unclassified Lachnocostridium* (*p* < 0.05), and *unclassified Prevotellaceae UCG-001* (*p* < 0.01) (Figure 3h). These suggested that IgAN induced disruptions in gut microbiota structure and diversity imbalances.

### IgAN-induced intestinal barrier damage and intestinal B-cell immune imbalance

**Figure 4.**
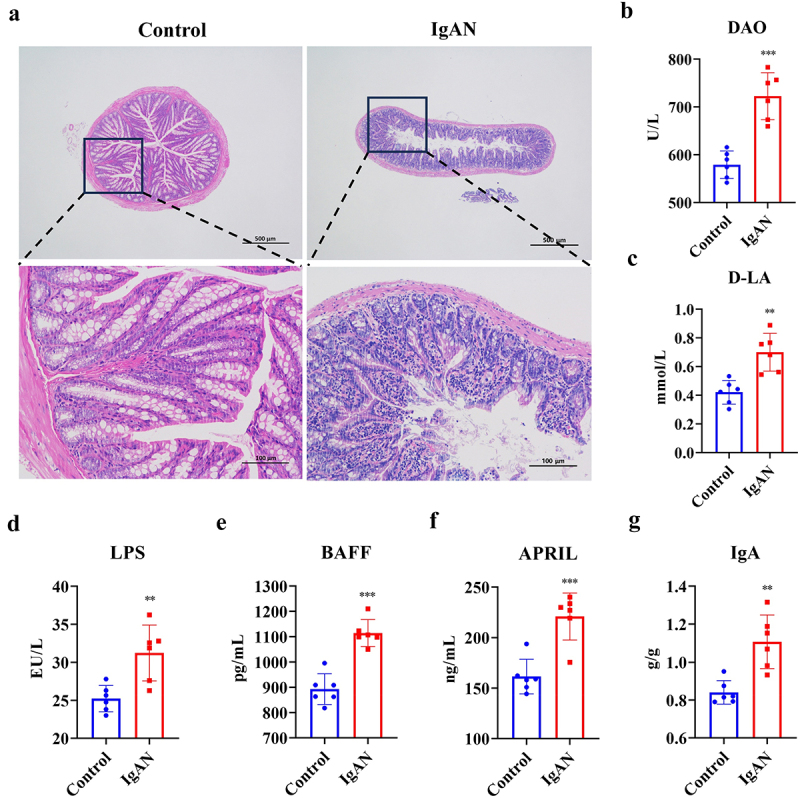
IgAN-induced intestinal barrier damage (IgAN vs control group). a H&E staining of colon tissue (scale bars = 100 μm and 50 μm, respectively, *n* = 3). b–d Measurement of indicators characterizing intestinal barrier permeability (DAO, D-LA, LPS) (*n* = 6). e–d Determination of serum B-cell activating factors (BAFF, APRIL) (*n* = 6). g Serum IgA level determination (*n* = 6). *Represented *p* < 0.05, **Represented *p* < 0.01, and ***Represented *p* < 0.001. Abbreviations: diamine oxidase (DAO), D-lactic acid (D-LA), lipopolysaccharides (LPS), B cell-activating factor (BAFF), and a proliferation-inducing ligand (APRIL).

H&E staining was used to evaluate the colonic integrity in various groups of treated mice to determine changes in intestinal permeability caused by IgAN (Figure 4a). The control group had intact mucosal epithelium, abundant glands in the lamina propria, densely and orderly arranged, and a clear muscular layer structure. Meanwhile, IgAN model mice exhibited loose glandular structures in the intestines, localized looseness, and thickening of the muscular layer, accompanied by minimal lymphocytic infiltration. Additionally, the assessment of intestinal permeability indicators (Figure 4b–d) revealed significant increases in serum levels of diamine oxidase (DAO) (*p* < 0.001), D-Lactic acid (D-LA) (*p* < 0.01), and LPS (*p* < 0.01) in the IgAN group, particularly in DAO levels, indicating an increase in intestinal permeability in IgAN model mice.

Gd-IgA1, an essential pathogenic factor in IgAN, results from abnormal glycosylation of IgA1 and was thought to originate from the intestine possibly.^23,24^ Intestinal B cells were the primary source of IgA production.^11,25^ This study elucidated IgAN’s immune imbalance by assessing B cell activation factors and IgA levels. The results found that the IgAN group exhibited significantly higher levels of B-cell activating factor (BAFF) (*p* < 0.001) and a proliferation inducing ligand (APRIL) (*p* < 0.001) compared to the control group. IgA levels were also abnormally elevated (*p* < 0.01), indicating that B cell overactivation leads to IgA homeostasis imbalance (Figure 4e–g). In summary, the disruption of the gut microbiota in IgAN model mice led to reduced intestinal barrier integrity and an imbalance in B cell secretion of IgA.

### COS remodeled microbiota dysbiosis in IgAN mice model

**Figure 5.**
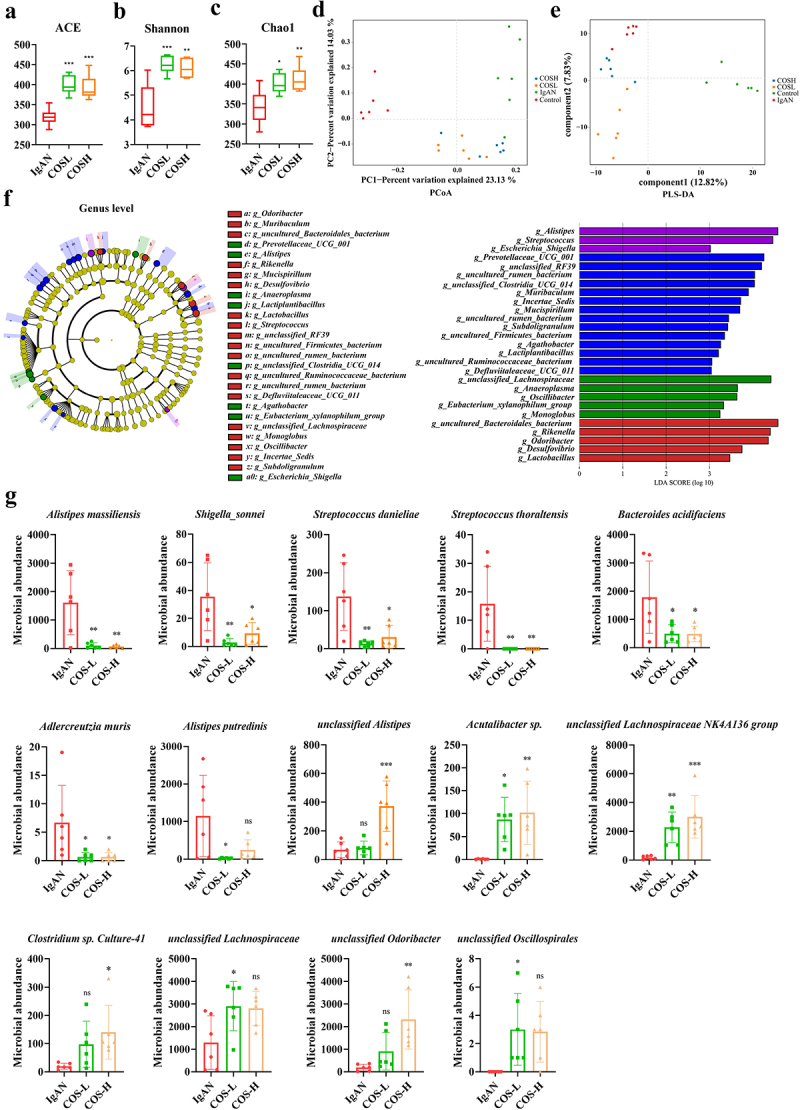
COS alleviated IgAN-induced gut microbiota dysbiosis (COS groups vs. IgAN group) (*n* = 6). a–c comparison of α-diversity indices of gut microbiota. d PCoA analysis of gut microbiota at the OTU level. e PLS-DA analysis of gut microbiota at the OTU level. f LEfSe analysis at the genus level and the score histograms. g identification of species-level differential taxa between IgAN, COS-L, and COS-H groups. Significance was obtained by comparison with the IgAN group. * represented *p* < 0.05, ** represented *p* < 0.01, *** represented *p* < 0.001 and ns represented *p* > 0.05.

To explore whether modulating the specific microbial community of IgAN could improve the progression, COS was applied for targeted regulation of the disordered microbial community in IgAN. The comparison of α-diversity revealed that the gut microbiota of the IgAN mice model showed an overall restoration with different doses of COS intervention. The ACE (COS-L, *p* < 0.001; COS-H, *p* < 0.001), Shannon (COS-L, *p* < 0.001; COS-H, *p* < 0.01), and Chao1 (COS-L, *p* < 0.05; COS-H, *p* < 0.01) indices were significantly increased (Figure 5a–c). β-diversity analysis (PCoA and PLS-DA) demonstrated that the gut microbiota following COS intervention exhibited notable alterations in comparison to the IgAN model group, exhibiting a shift toward the control group (Figure 5d,e).

Analysis of the gut microbiota composition revealed that the enriched microbiota differed after intervention with various doses of COS, with the COS-L group mainly enriching *unclassified Lachnospiraceae*, *Anaeroplasma*, *Oscillibacter*, *Eubacterium xylanophilum group*, and *Monoglobus*. Meanwhile, the COS-H group primarily enriched the *uncultured Bacteroidales bacterium*, *Rikenella*, *Odoribacter*, and *Lactobacillus* (Figure 5f). Both COS-L and COS-H effectively suppressed the significantly increased microbial abundances in the IgAN model, including *Alistipes massiliensis* (COS-L, *p* < 0.01; COS-H, *p* < 0.01), *Shigella sonnei* (COS-L, *p* < 0.01; COS-H, *p* < 0.05), *Streptococcus Danieline* (COS-L, *p* < 0.01; COS-H, *p* < 0.05), *Streptococcus thoraltensis* (COS-L, *p* < 0.01; COS-H, *p* < 0.01), *Bacteroides acidifaciens* (COS-L, *p* < 0.05; COS-H, *p* < 0.05), and *Adlercreutzia muris* (COS-L, *p* < 0.05; COS-H, *p* < 0.05) (Figure 5g). Thus, a reverse balancing effect on the gut microbiota of the IgAN model was achieved. Taken together, these findings suggested that COS, as a functional oligosaccharide with microbiota-targeting regulatory effects, can enhance the diversity of the gut microbiota in the IgAN model and effectively reverse the dysregulation of the specific microbial community, resulting in a transition of the microbial structure toward a healthier state.

### Targeted microbial regulation attenuated intestinal barrier damage and balanced intestinal B-cell immunity

**Figure 6.**
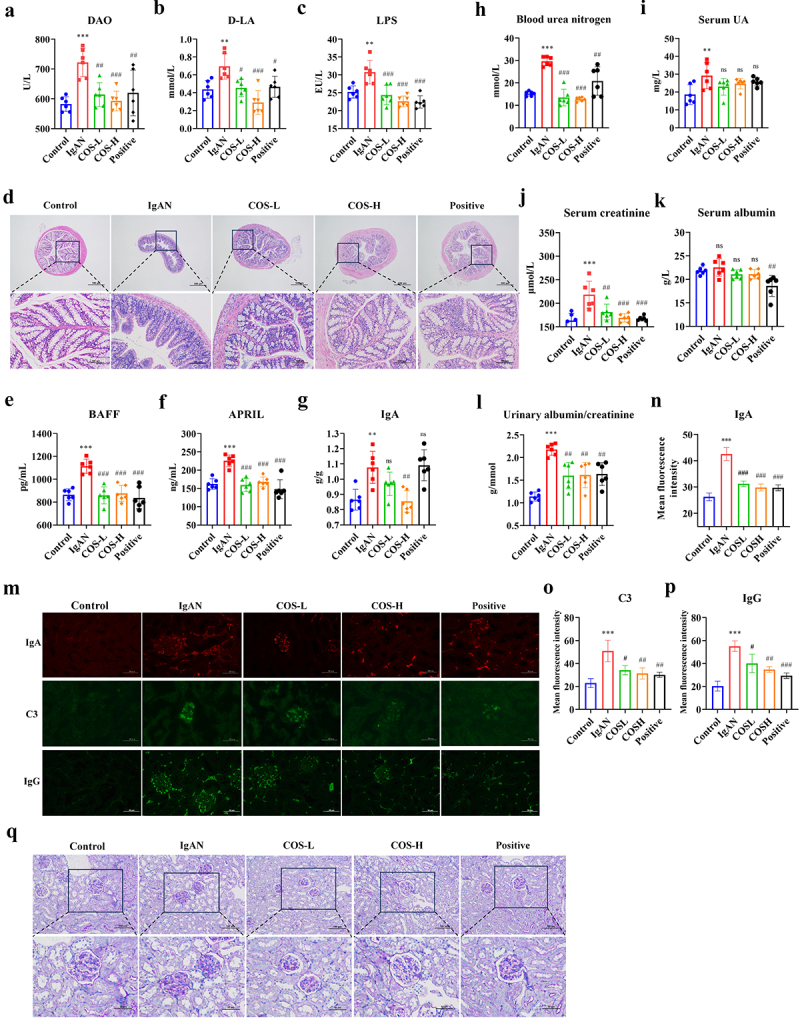
COS improved IgAN-induced intestinal barrier damage and renal dysfunction (COS groups and positive control group vs. IgAN group). a–c assessment of intestinal barrier permeability indicators (DAO, D-LA, LPS) (*n* = 6). d H&E staining of colon tissue (scale bars = 100 μm and 50 μm, respectively) (*n* = 3). e–f assessment of serum B-cell activating factors (BAFF, APRIL) (*n* = 6). g determination of serum IgA levels (*n* = 6). h–l assessment of renal function indicators (*n* = 6). M-P glomerular IgA, C3, and IgG immunofluorescence and quantitative analysis (scale bar = 50 μm, *n* = 3). Q PAS staining of the kidney (scale bars = 100 μm and 50 μm, respectively, *n* = 3). Significance * and # were obtained by comparison with the control and IgAN groups, respectively. * (#) represented *p* < 0.05, ** (##) represented *p* < 0.01, *** (###) represented *p* < 0.001 and ns represented *p* > 0.05.

Further, the effects of modulating the specific microbial community on intestinal barrier and mucosal immunity were explored. Results indicated that the COS intervention groups significantly improved intestinal permeability indicators including DAO (COS-L, *p* < 0.01; COS-H, *p* < 0.001), D-LA (COS-L, *p* < 0.05; COS-H, *p* < 0.001), and LPS (COS-L, *p* < 0.001; COS-H, *p* < 0.001) (Figure 6a–c). The improvement effects on D-LA and LPS were dose-dependent, with the COS-H group showing the best improvement. The positive control group also showed improved intestinal damage caused by the IgAN model. The H&E staining also revealed that COS intervention resulted in clear and complete colonic structures, with regularly arranged intestinal glands, trending toward normalization (Figure 6d).

Furthermore, the COS intervention alleviated the IgA imbalance caused by B cell hyperactivation in the IgAN model. COS intervention significantly suppressed the serum levels of BAFF (COS-L, *p* < 0.001, COS-H, *p* < 0.001) and APRIL (COS-L, *p* < 0.001, COS-H, *p* < 0.001) in mice. The IgA levels were attenuated in the COS-H group (*p* < 0.01), with no significant difference in the COS-L group (*p* > 0.05) (Figure 6e–g). Therefore, targeting adjustments to the specific microbial community in IgAN can inhibit overexpression of APRIL and BAFF, thus exerting an immunomodulatory effect on IgA.

### COS intervention delayed IgAN-induced renal injury and restores renal function

Administration of COS ultimately led to improvements in renal function indices. Notably, COS intervention most effectively lowered BUN (COS-L, *p* < 0.001; COS-H, *p* < 0.001), also showing significant improvements in Scr (COS-L, *p* < 0.01; COS-H, *p* < 0.001), and ACR (COS-L, *p* < 0.01; COS-H, *p* < 0.01) levels (Figure 6h–l). Renal immunofluorescence staining and PAS staining revealed that COS intervention reduced IgA (COS-L, *p* < 0.001; COS-H, *p* < 0.001), C3 (COS-L, *p* < 0.05; COS-H, *p* < 0.01) and IgG deposition (COS-L, *p* < 0.05; COS-H, *p* < 0.01) (Figure 6m–p) and improved glomerular mesangial proliferation and matrix thickening, most effectively in the COS-H group (Figure 6q). Consequently, modulating the specific microbial community in IgAN ameliorated its progression and reduced renal function impairment.

### Renal injury induced by transplantation of fecal microbiota from IgAN patients

**Figure 7.**
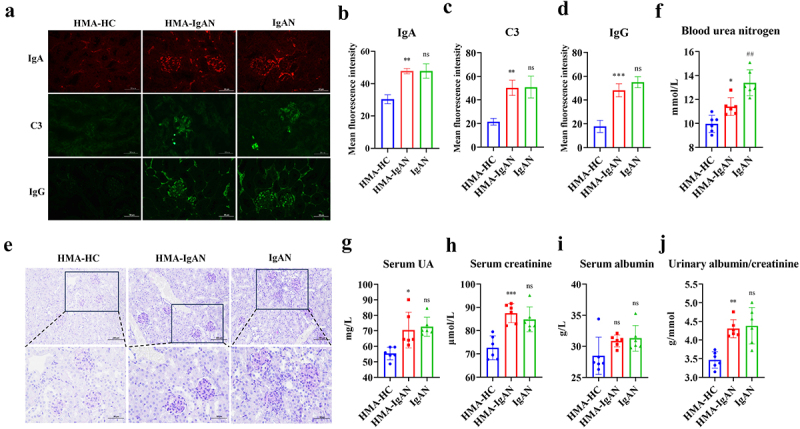
Identification of the HMA-IgAN model. (HMA-HC and IgAN groups vs. HMA-IgAN group). a–d glomerular IgA, C3, and IgG immunofluorescence and quantitative analysis (scale bar = 50 μm, *n* = 3). e PAS staining of the kidney (scale bars = 100 μm and 50 μm, respectively, *n* = 3). f–j assessment of renal function indicators (*n* = 6). The significance of the HMA-IgAN group was obtained by comparison with the HMA-HC group, and the significance of the IgAN group was obtained by comparison with the HMA-IgAN group. * represented *p* < 0.05, ** represented *p* < 0.01, *** represented *p* < 0.001 and ns represented *p* > 0.05.

The HMA-IgAN mice model was used to address the differences in microbiota between mice models and humans, verifying the impact of IgAN-induced human gut microbiota evolution on mice renal function. Renal immunofluorescence demonstrated glomerular IgA (*p* < 0.01), C3 (*p* < 0.01), and IgG (*p* < 0.001) deposition in HMA-IgAN mice akin to that observed in IgAN model mice (Figure 7a–d). PAS staining showed significant glomerular mesangial proliferation with inflammatory infiltration in HMA-IgAN mice, resembling the IgAN model group (Figure 7e). HMA-IgAN mice exhibited renal injury similar to IgAN models, with significant increases in BUN (*p* < 0.05), UA (*p* < 0.05), Scr (*p* < 0.001), and ACR (*p* < 0.01) compared to the HMA-HC group (Figure 7f–j). These findings suggested that the gut microbiota under IgAN could induce the successful construction of the IgAN mice model and cause renal injury in the host, comparable to the damage observed in IgAN mice model.

### The HMA-IgAN mice model recapitulated gut microbiota dysregulation

**Figure 8.**
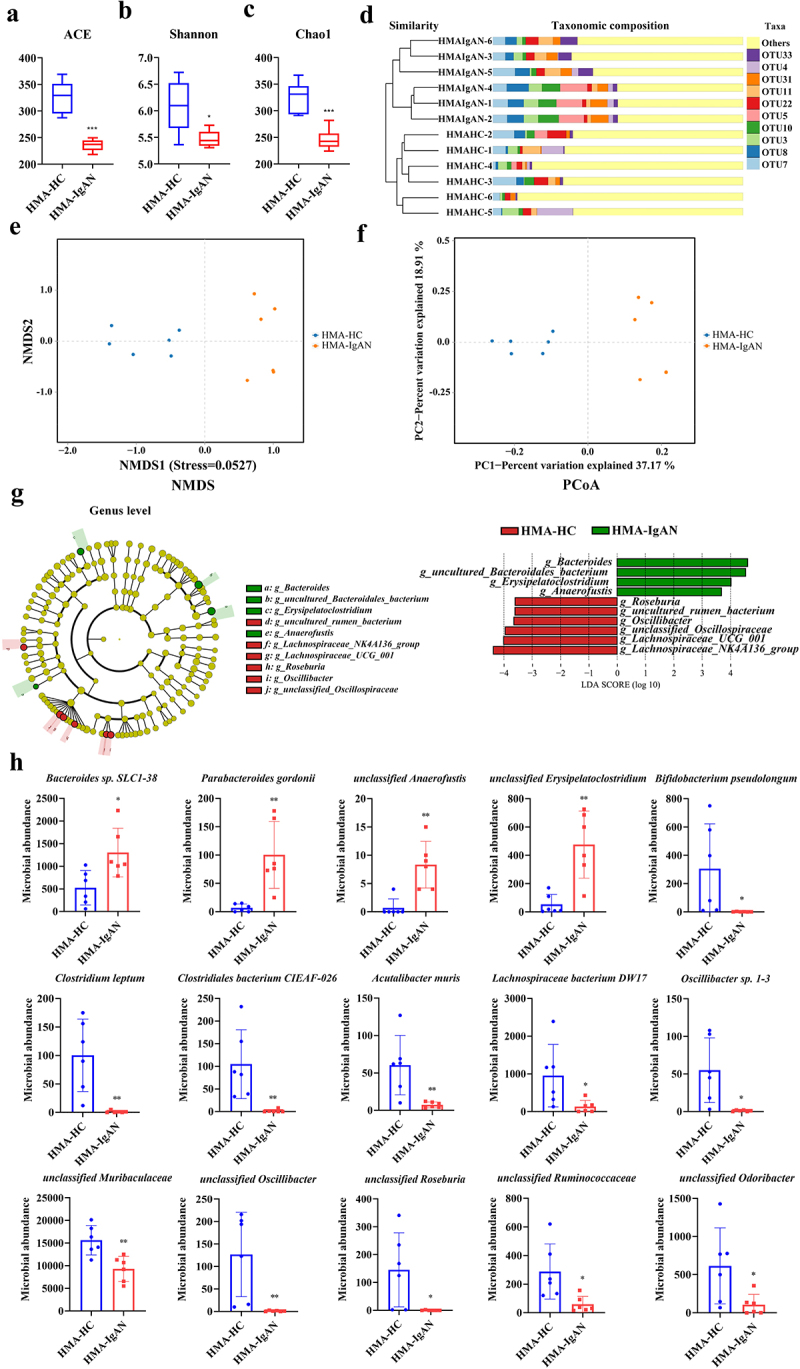
Disruption and diversity imbalance in gut microbiota caused by the HMA-IgAN mice model (HMA-IgAN group vs. HMA-HC group) (*n* = 6). a–c comparison of α-diversity in gut microbiota. d UPGMA analysis of the dendrogram structure between the two groups of samples. e NMDS analysis of gut microbiota at the OTU level. f PCoA analysis of gut microbiota at the OTU level. g LEfSe analysis at the genus level and the score histograms. h identification of species-level differential taxa between the two groups. * represented *p* < 0.05, ** represented *p* < 0.01, and *** represented *p* < 0.001.

Compared to the HMA-HC group, the diversity indices ACE (*p* < 0.001), Shannon (*p* < 0.05), and Chao1 (*p*  < 0.001) of the gut microbiota in the HMA-IgAN group significantly decreased (Figure 8a–c). β-diversity analysis using UPGMA, NMDS, and PCoA showed distinct differentiation between the clusters of the two groups (Figure 8d–f). Significant differences were also observed in the microbiota characteristics of the two groups. Bacteroides were more abundant in the HMA-IgAN mice. At the genus level, HMA-IgAN mice showed an increase in *Bacteroides, uncultured Bacteroidales bacterium, Erysipelatoclostridium*, and *Anaerofustis* (Figure 8g). At the species level, representative microbiota in the HMA-IgAN group included *Bacteroides sp. SLC1–38* (*p* < 0.05), *Parabacteroides gordonii* (*p*  < 0.01), *unclassified Anaerofustis* (*p*  < 0.01), and *unclassified Erysipelatoclostridium* (*p* < 0.01). However, the abundances of *Bifidobacterium pseudolongum* (*p* < 0.05), *Clostridium leptum* (*p* < 0.01), *Acutalibacter muris* (*p* < 0.01), *unclassified Oscillibacter* (*p* < 0.01), *unclassified Roseburia* (*p* < 0.05), *unclassified Ruminococcaceae* (*p* < 0.05), and *unclassified Odoribacter* (*p* < 0.05) were significantly reduced (Figure 8h). Therefore, the HMA-IgAN mice model can induce disturbances in the diversity of the gut microbiota (ACE, Shannon, and Chao1 indices) as well as alterations in the microbial community.

### The HMA-IgAN mice model induced intestinal barrier and B-cell immune imbalance

**Figure 9.**
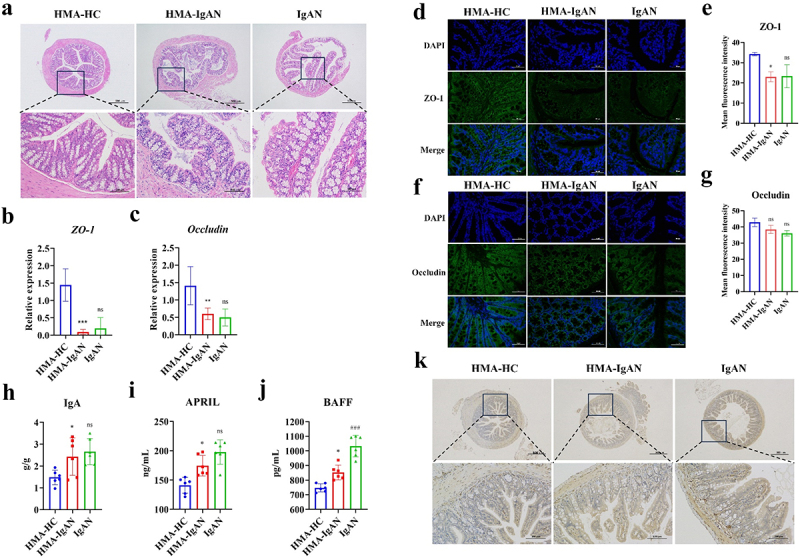
HMA-IgAN model induced intestinal barrier damage (HMA-HC and IgAN groups vs. HMA-IgAN group). a H&E staining of the colon (scale bars = 100 μm and 50 μm, respectively, *n* = 3). b–c assessment of ileal tight junction proteins (*ZO-1*, *Occludin*) gene expression levels (*n* = 6). d–e immunofluorescence and quantitative analysis of ileal ZO-1 (scale bar = 50 μm, *n* = 3). f–g immunofluorescence and quantitative analysis of ileal occludin (scale bar = 50 μm, *n* = 3). h determination of serum IgA levels (*n* = 6). i–j assessment of serum B-cell activating factors (APRIL, BAFF) (*n* = 6). K immunohistochemical analysis of ileal IgA (scale bars = 500 μm and 100 μm, respectively, *n* = 3). The significance of the HMA-IgAN group was obtained by comparison with the HMA-HC group, and the significance of the IgAN group was obtained by comparison with the HMA-IgAN group. * represented *p* < 0.05, ** represented *p* < 0.01, *** represented *p* < 0.001 and ns represented *p* > 0.05.

The intestinal barrier function and B-cell homeostasis were assessed in HMA-IgAN mice. Colonic H&E staining indicated significant impairment of the intestinal barrier in HMA-IgAN mice, with worsened tight junctions and epithelial structural integrity. In contrast, the HMA-HC group displayed clear and intact intestinal structures (Figure 9a). Moreover, the gene expression levels of intestinal tight junction proteins *ZO-1* (*p* < 0.001) and *Occludin* (*p* < 0.01) in HMA-IgAN mice were significantly lower than those in the HMA-HC group. Whereas comparisons between the HMA-IgAN and IgAN groups revealed no significant differences in ZO-1 (*p* > 0.05) and Occludin (*p* > 0.05) (Figure 9b,c), indicating similar degree of intestinal permeability disruption across the two model groups. Immunofluorescence results showed a significant decrease in ZO-1 (*p* < 0.05) expression (Figure 9d,e), with no considerable inhibition observed for Occludin (*p* > 0.05) (Figure 9f,g).

The HMA-IgAN mice model also exhibited dysregulated B-cell homeostasis. Serum IgA (*p* < 0.05), APRIL (*p* < 0.05), and BAFF (*p* < 0.05) levels were abnormally elevated compared to the HMA-HC group, while the IgAN model showed a greater degree of disorder (Figure 9h-j). Studies reported that Gd-IgA1 originated from Peyer’s patches at the terminal ileum, suggesting a close link between intestinal IgA homeostasis and IgAN. Ileal IgA immunohistochemistry was performed in this study, showing a notable increase in IgA positivity in both HMA-IgAN and IgAN model groups, suggesting massive IgA production and B cell hyperactivation (Figure 9k). Consequently, the dysregulated gut microbiota from IgAN patients leads to intestinal barrier damage, increased intestinal permeability, and imbalanced intestinal B cell immunity in mice models.

### COSF increased gut microbiota diversity and maintained intestinal homeostasis in the HMA-IgAN mice model

**Figure 10.**
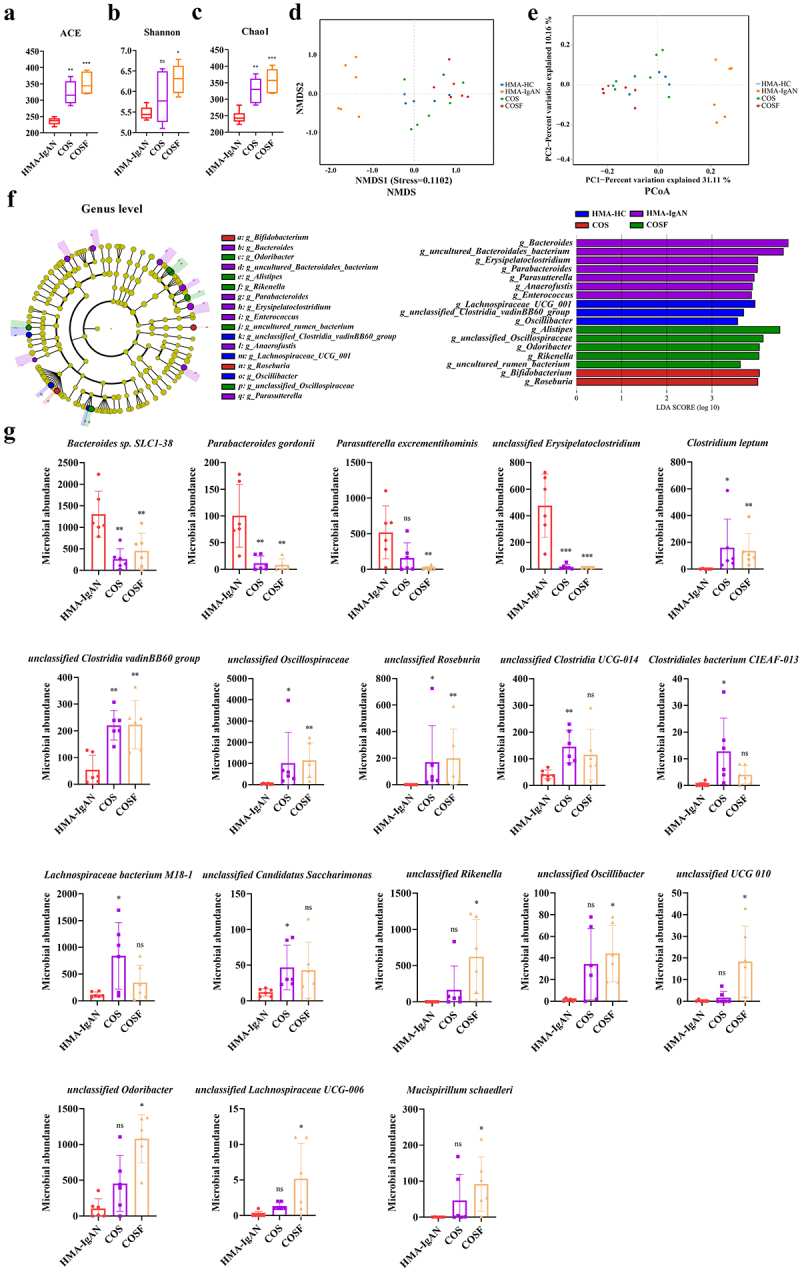
Restoration of gut microbiota balance in the HMA-IgAN mice model by COS and COSF (COS and COSF groups vs. HMA-IgAN group) (*n* = 6). a-c comparison of α-diversity in gut microbiota. d NMDS analysis of gut microbiota at the OTU level. e PCoA analysis of gut microbiota at the OTU level. f LEfSe analysis at the genus level and the score histograms. g identification of species-level differential taxa between the HMA-IgAN group and intervention groups. Significance was obtained by comparison with the HMA-IgAN group. * represented *p* < 0.05, ** represented *p* < 0.01, *** represented *p* < 0.001 and ns represented *p* > 0.05.

To investigate whether targeted microbial modulation could improve the progression of the HMA-IgAN mice model, the experiment employed COS and COSF for intervention. The results showed that COS intervention significantly increased the α-diversity indices ACE and Chao1 of the gut microbiota in HMA-IgAN mice (*p* < 0.001, *p* < 0.01), while the Shannon index exhibited no significant difference (*p* > 0.05) (Figure 10a–c). COSF significantly promoted the ACE (*p* < 0.001), Shannon (*p* < 0.05) and Chao1 (*p* < 0.001) indices (Figure 10a-c), which resulted in an increase in the richness of the gut microbiota. The clustering results showed that samples in the HMA-IgAN group were distinctly separated from those of the control and intervention groups, with intervention group samples tending to be similar to the control group (Figure 10d,e).

LEfSe analysis was used to compare the differential microbiota among the HMA-HC group, HMA-IgAN group, COS intervention group, and COSF intervention group, identifying the differential microbiota of each group (Figure 10f). At the genus level, COS could enrich *Bifidobacterium* and *Roseburia*. The enriched microbiota in the COSF group included *Alistipes*, *unclassified Oscillospiraceae*, *Odoribacter*, *Rikenella*, and *uncultured rumen bacterium*. At the species level (Figure 10g), COS and COSF effectively inhibited the proliferation of *Bacteroides sp. SLC1–38* (COS, *p* < 0.01; COSF, *p* < 0.01), *Parabacteroides gordonii* (COS, *p* < 0.01; COSF, *p* < 0.01), and *unclassified Erysipelatoclostridium* (COS, *p* < 0.001; COSF, *p* < 0.001) within the model group and facilitated the enrichment of *Clostridium leptum* (COS, *p* < 0.05; COSF, *p* < 0.01), *unclassified Clostridia vadinBB60 group* (COS, *p* < 0.01; COSF, *p* < 0.01), *unclassified Oscillospiraceae* (COS, *p* < 0.05; COSF, *p* < 0.01), and *unclassified Roseburia* (COS, *p* < 0.05; COSF, *p* < 0.01). Combining the microbial changes after COS intervention in the IgAN model group, it can be concluded that COS and COSF can balance the dysbiosis of the specific microbial community in the two models (Table S3).

### COSF intervention modulated impaired intestinal barrier and dysregulated intestinal B-cell immunity in HMA-IgAN mice model

**Figure 11.**
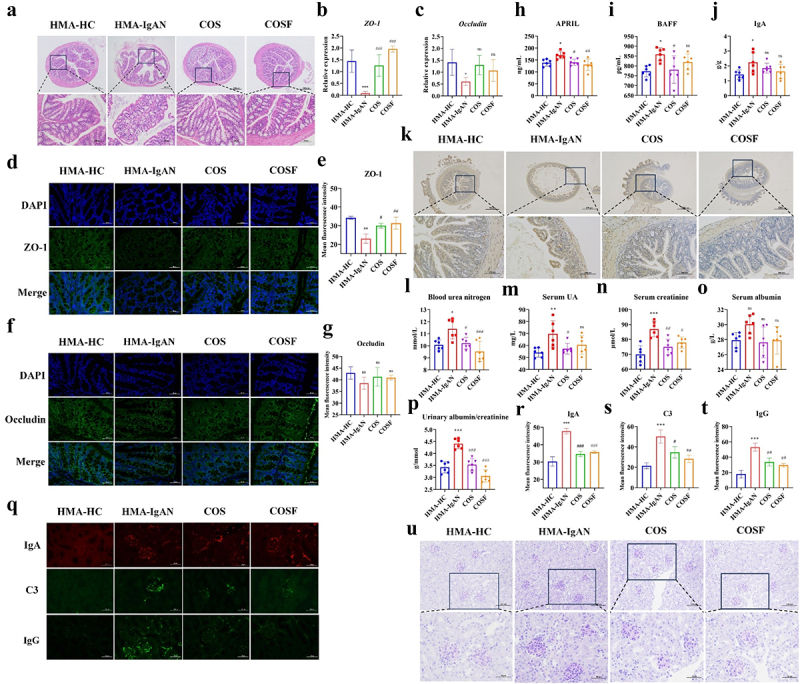
Amelioration of intestinal barrier and renal function in the HMA-IgAN model by COS and COSF. a H&E staining of the colon (scale bars = 100 μm and 50 μm, respectively, *n* = 3). b-c assessment of ileal tight junction proteins (*ZO-1*, *Occludin*) gene expression levels (*n* = 6). d-e immunofluorescence and quantitative analysis of ileal ZO-1 (scale bar = 50 μm, *n* = 3). f-g immunofluorescence and quantitative analysis of ileal occludin (scale bar = 50 μm, *n* = 3). h-i assessment of serum B-cell activating factors (APRIL, BAFF) (*n* = 6). j determination of serum IgA levels (*n* = 6). k immunohistochemical analysis of ileal IgA (scale bars = 500 μm and 100 μm, respectively, *n* = 3). l-p assessment of renal function indicators (*n* = 6). Q-T glomerular IgA, C3, and IgG immunofluorescence and quantitative analysis (scale bar = 50 μm, *n* = 3). U PAS staining of the kidney (scale bars = 100 μm and 50 μm, respectively, *n* = 3). Significance * and # were obtained by comparison with the HMA-HC and HMA-IgAN groups, respectively. * (#) represented *p* < 0.05, ** (##) represented *p* < 0.01, *** (###) represented *p* < 0.001 and ns represented *p* > 0.05.

COS and COSF intervention restored the integrity of the mice’s intestinal structure (Figure 11a). COS intervention significantly increased the gene expression levels of intestinal tight junction proteins *ZO-1* (*p* < 0.001) and *Occludin* (*p* < 0.05) in mice. The COSF group exhibited more effective recuperative capability for *ZO-1* (*p* < 0.001), showing a trend of increased gene expression for *Occludin*, although not statistically significant (*p* > 0.05) (Figure 11b,c). Additionally, the immunofluorescence expression levels of ZO-1 and Occludin in the ileum were consistent with the gene expression results, with COS (*p* < 0.05) and COSF (*p* < 0.01) intervention having a more pronounced promoting effect on ZO-1 (Figure 11d,e), but not significantly affecting the expression of Occludin (COS, *p* > 0.05; COSF, *p* > 0.05) (Figure 11f,g).

Measurement of serum B cell activating factor levels showed that COS intervention effectively suppressed APRIL and BAFF in the HMA-IgAN group (COS, *p* < 0.05; COSF, *p* < 0.01) (Figure 11h,i), positively restoring B cell homeostasis. COSF intervention notably inhibited APRIL (*p* < 0.05), but had no significant effect on BAFF (*p* > 0.05) (Figure 11h,i). No significant modulation of serum IgA levels in HMA-IgAN mice model by COS and COSF interventions was found (COS, *p* > 0.05, COSF, *p* > 0.05) (Figure 11j). Moreover, COS intervention mitigated the expression of ileal IgA in HMA-IgAN mice model mice, and COSF group showed an even better improvement effect (Figure 11k). Overall, targeted modulation of the specific microbial community by COS and COSF can ameliorate intestinal barrier damage and intestinal B cell immune dysregulation caused by gut microbiota disorder.

### COSF improved renal function repair in HMA-IgAN mice model

COS effectively improved renal function in the HMA-IgAN mice model (Figure 11l–p), significantly reducing UA (*p* < 0.05), Scr (*p* < 0.01), BUN (*p* < 0.05), and ACR (*p* < 0.001) levels. The COSF group exhibited significant improvement in BUN (*p* < 0.001), Scr (*p* < 0.05), and ACR (*p* < 0.001) levels, especially in reducing ACR, but had no significant effect on UA (*p* > 0.05) and BLA (*p* > 0.05). Glomerular immunofluorescence staining and PAS staining results showed that COS and COSF intervention significantly reduced IgA (COS, *p* < 0.001; COSF, *p* < 0.001), C3 (COS, *p* < 0.05; COSF, *p* < 0.01) and IgG (COS, *p* < 0.01; COSF, *p* < 0.01) deposition (Figure 11q–t), and had a noticeable inhibitory effect on mesangial proliferation and matrix expansion (Figure 11u), with COSF group showing more significant improvement in glomerular mesangial area proliferation. Therefore, COSF intervention could enhance renal function through targeted modulation of the specific microbial community within the HMA-IgAN mice model.

### Association analysis of microbiota with intestinal barrier, B-cell immunity and renal function indices

**Figure 12.**
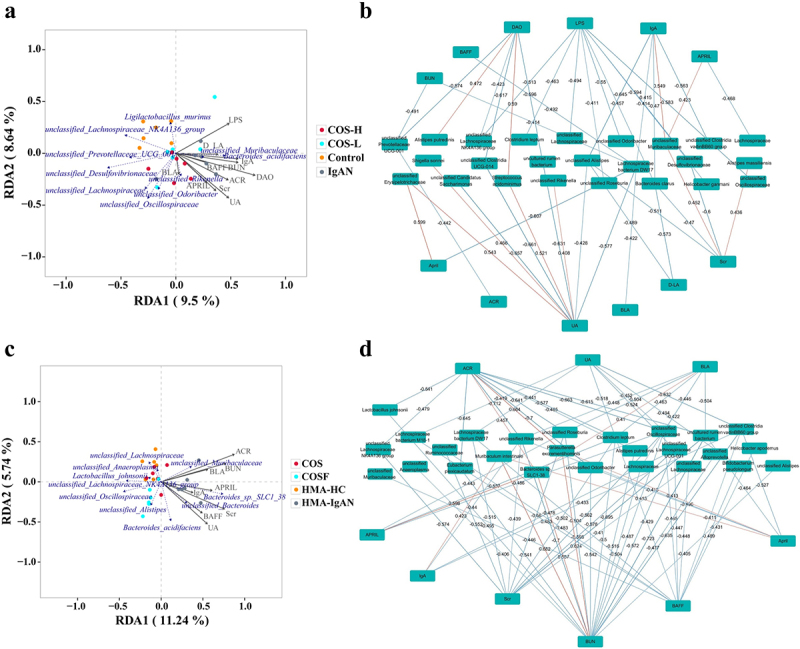
Correlation analysis between gut microbiota, renal function, and intestinal barrier indicators (*n* = 6). a, c RDA analysis of species-level gut microbiota, renal function indices, and intestinal barrier indicators with samples across groups. b, d network interactions between species-level gut microbiota, renal function, and intestinal barrier indicators. Green rectangles represent species and indicators, blue lines indicate negatively correlated (np) species, and orange lines indicate positively correlated (pp) species. a, b represents COS intervention in IgAN animal experiments; c, d represents COS and COSF interventions in HMA-IgAN animal experiments.

To analyze the role of the specific microbial community in intestinal barrier function, B-cell immunity, and renal function, network correlation analyses were performed. Redundancy analysis (RDA) results showed that renal function indicators (UA, BUN, ACR, and Scr), intestinal barrier indicators (DAO, LPS, and D-LA), and IgA-related immune indicators (IgA, APRIL, BAFF) closely reflected the severity of IgAN progression. UA, ACR, Scr, BUN, DAO, and IgA were particularly indicative, with APRIL and BAFF being more pronounced in the HMA-IgAN group. Specific microbiota, such as *unclassified Muribaculaceae*, *Bacteroides acidifaciens*, *Bacteroides sp. SLCl-38*, and *unclassified Bacteroides* were positively correlated with IgAN, while the *unclassified Lachnospiraceae NK4A136 group*, *unclassified Oscillospiraceae*, *unclassified Prevotellaceae UCG-001*, and *Lactobacillus johnsonii* were negatively correlated (Figure 12a,c).

In the IgAN model, microbiota like *unclassified Erysipelotrichaceae, Streptococcus acidominimus*, and *Shigella sonnei* showed significant positive correlations with poor renal function and excessive immune response, whereas *unclassified Alistipes* and *Clostridia UCG-014* showed negative correlations (*p* < 0.05). Similar patterns were observed in the HMA-IgAN model, with *Bacteroides sp. SLC1–38* and *Muribaculum intestinale* showing positive correlations and *Bifidobacterium pseudolongum* and *unclassified Ruminococcaceae* showing negative correlations (Figure 12b,d; Table S4).

The specific microbial community enriched by COS and COSF interventions significantly correlated with favorable IgAN outcomes. For example, *Clostridium leptum* exhibited negative correlations with Scr, UA, BUN, ACR, and APRIL (*p* < 0.05, *r* < −0.5). Other microorganisms, including *unclassified Lachnospiraceae NK4A136 group, unclassified Clostridia vadinBB60 group, unclassified Odoribacter, unclassified Rikenella*, and *unclassified Roseburia*, were also significantly and positively associated with a favorable prognosis (gut barrier restoration and immune improvement) in IgAN (Figure 12b,d; Table S5).

### Discussion

Researches on the relationship between the gut microbiota and IgAN are gaining attention. However, the mechanism by which the gut microbiota affects IgAN and its potential as an effective intervention target for IgAN remains uncertain. This study investigated the interrelation and interactions between gut microbiota and IgAN. It revealed that changes in microbiota related to the intestinal barrier and mucosal immunity play an important role in the development and progression of IgAN. Furthermore, COS and COSF, which have microbiota-targeting effects, could effectively reduce the pathological phenotype of IgAN.

Gut microbiota dysbiosis and the resulting metabolic disturbances have been shown to be closely related to the progression of chronic kidney disease (CKD), including IgAN, membranous nephropathy (MN), idiopathic membranous nephropathy (IMN), and other kidney injury-related diseases.^26–28^ MN was reported to reduce abundances of *Lactobacillus johnsonii*, *Lactobacillus murinus*, *Lactobacillus vaginalis*, *Lactobacillus reuteri*, and *Bifidobacterium animalis*, which could regulate MN through the aryl hydrocarbon receptor (AhR) pathway.^29^ Patients with IMN exhibited a reduction in *Enterococcus faecium* and *Bacteroides*, along with an increase in the abundance of unidentified *Enterobacteriaceae*, *Bifidobacterium*, and *Romboutsia*, which led to a decrease in tryptophan metabolism.^26^ Clinical studies showed that patients with IgAN showed favorable outcomes with beneficial improvements in clinical indicators after treatment with FMT, indicating the important role of gut microbiota in the development of IgAN.^30,31^ However, most studies were limited to clinical observations, leading to some contradictory findings (e.g. *Bacteroides*, *Ruminococcus*, *Prevotella*, *Faecalibacterium* changes in IgAN).^29,32^ Our study found reduced gut microbiota diversity and significant compositional differences in IgAN patients compared to healthy individuals. Notably, the abundance of *Shigella*, *Ruminococcus*, *Erysipelotrichaceae Clostridium*, and *Eggerthella* increased, while *Bifidobacterium*, *Oscillospira*, and *Roseburia* decreased. We further identified the key microbial community through animal models and found that changes in microbes associated with the intestinal barrier and mucosal immunity were critical for IgAN progression. Due to differences in gut microbiota composition between animal models and humans, this study utilized HMA-IgAN mice models to better understand the causal links between gut microbiota and IgAN development. Both IgAN and HMA-IgAN mice models exhibited a significant decrease in gut microbiota diversity and an imbalance in microbiota associated with the intestinal barrier and mucosal immunity. The IgAN models showed a significant rise in microbiota including *Shigella sonnei*, *Streptococcus danieliae*, *Streptococcus thoraltensis*, and *Desulfovibrio fairfieldensis*. *Shigella sonnei* can inject effector proteins into host cells via the Type III secretion system, disrupting the host’s cytoskeleton, leading to loss of intracellular barrier function, and aggravating inflammation.^33,34^ *Streptococcus danieliae* and *Streptococcus thoraltensis*, identified as infection-causing bacteria, were considered to be associated with inflammatory diseases.^35,36^ *Desulfovibrio fairfieldensis* can produce harmful metabolites such as hydrogen sulfide, leading to compromised tight junctions and intestinal barrier function.^37,38^

Transplantation of fecal microbiota from IgAN patients induced an IgAN phenotype in pseudo-germ-free mice, indicating gut microbiota alteration was an essential driver of IgAN pathogenesis. Zhu et al. found that IgAN-FMT as well as IgAN-treated-FMT mice induced elevated blood creatinine and urinary albumin levels and resulted in immunodeposition of renal IgA and C3, whereas FMT mice performed using MN patients and HC population did not show the above-mentioned impairments.^39^ The results of our study showed that HMA-IgAN mice also exhibited dysregulation of microbiota associated with the gut barrier and mucosal immunity, notably a marked decrease in *Bifidobacterium pseudolongum*, *Clostridium leptum*, *unclassified Oscillibacter*, *unclassified Roseburia*, *unclassified Ruminococcaceae*, and *unclassified Odoribacter*. The probiotic *Bifidobacterium pseudolongum* had the ability of producing SCFAs, particularly butyrate, which can activate G-protein-coupled receptors on the surface of epithelial cells, and enhance the intestinal barrier function.^40,41^ Furthermore, *Bifidobacterium pseudolongum* can enhance Th1 cell activation and effector T cell function.^42^ Studies reported that *Clostridium leptum* can ferment flaxseed polysaccharides to produce beneficial metabolites that alleviate obesity, and it can induce the generation of IL-10 regulatory B cells, thus mitigating airway inflammation in asthma patients.^43,44^ Other microbial groups at the genus level, such as *Roseburia* (related to butyrate production),^45^ *Ruminococcaceae* (capable of cellulose degradation),^46^ *Oscillibacter*,^47^ and *Odoribacter*,^48^ were considered to maintain intestinal epithelial cell health as well as intestinal immune homeostasis through the production of beneficial metabolites (SCFAs). Therefore, alterations in specific microbial community associated with the intestinal barrier and mucosal immunity were key regulators in IgAN development and progression.

Alterations in gut microbiota caused by IgAN disrupted IgA-associated intestinal B cell immunity and damaged the gut barrier. IgAN models showed increased serum indicators (DAO, D-LA, LPS) and decreased expression of intestinal tight junction proteins (ZO-1 and Occludin) in HMA-IgAN mice models, particularly ZO-1, indicating impaired intestinal barrier integrity. The integrity of the intestinal structure relies heavily on tight junction proteins, with ZO-1 crucial for mucosal repair.^49,50^ Intestinal barrier damage was closely related to imbalanced intestinal mucosal immunity. IgA, the most abundant immunoglobulin produced by the mucosal immune system, interacted with gut microbiota and contributed to autoimmune diseases.^51^ Studies reported that IgAN patients exhibited elevated levels of IgA, BAFF, and APRIL, suggesting a B cell homeostasis imbalance.^52^ Excessive IgA production in IgAN primarily occurs in B cells within the intestinal lamina propria. This study further examined IgAN’s impact on B cell activity and IgA generation, finding overexpression of B cell activation factors and increased IgA secretion. There was a kidney-gut crosstalk in IgAN, with changes in the specific microbial community affecting the homeostasis of the intestinal barrier and B cell immunity.

CKD led to the gradual loss of kidney function, ultimately progressing to ESRD, with IgAN being one of the major contributing factors. Renal replacement therapies in ESRD patients, such as hemodialysis (HD) and peritoneal dialysis (PD), can also modulate the gut microbiota. For example, studies have shown that in HD patients, the abundance of *Firmicutes* significantly decreases, while no significant differences were found in the families associated with uremic toxin production, including *Bifidobacteriaceae*, *Clostridiaceae*, *Enterobacteriaceae*, and *Lactobacillaceae*.^27^ This study proposed a strategy to improve IgAN by targeting and regulating microbiota associated with the intestinal barrier and mucosal immunity. Recent research highlighted the benefits of natural products such as polysaccharides, traditional Chinese medicine extracts, and probiotics in alleviating gut microbiota dysbiosis in chronic kidney disease.^53,54^ For example, Astragalus membranaceus, Salvia miltiorrhiza, and Rehmannia glutinosa regulated diabetic nephropathy by increasing the abundance of *Akkermansia*, *Lactobacillus*, *Ruminococcaceae UCG-014*, *Prevotellaceae NK3B31 group*, and *Lachnospiraceae UCG-001*, and by reducing serum uremic toxin (indoxyl-sulfate and *p*-cresol sulfate) levels.^55,56^ Natural polysaccharides, like the prebiotic COS, are increasingly used for intestinal protection. Additionally, supplementation with specific gut microbiota can improve the progression of CKD. Miao et al.^57^ found that the abundance of *Lactobacillus johnsonii* decreased as CKD progresses, while supplementation with *L. johnsonii* increased serum IAld levels and inhibited AHR signaling, thereby improving renal pathology. Our study explored the effects of COS and COSF on IgAN progression by modulating gut microbiota and intestinal functions. Administration of COS or COSF to IgAN and HMA-IgAN mice restored α-diversity and balanced the specific microbial community related to the intestinal barrier and mucosal immunity. For example, decreasing the abundance of *Shigella sonnei, Streptococcus danieliae, Streptococcus thoraltensis*, while increasing beneficial bacteria such as *Clostridium leptum*, *Lachnospiraceae NK4A136 group*, *Oscillospirales*, and *Odoribacter*. These beneficial bacteria enhance intestinal barrier function, primarily through their production of SCFAs.^58^ SCFA levels were reported diminished in IgAN patients, and supplementation with SCFAs-producing probiotics could mitigate IgAN pathology. Tan et al. verified that *Bifidobacteria* and their metabolites (SCFAs) could alleviate IgAN progression by inhibiting the NLRP3/ASC/Caspase 1 pathway.^59^ Additionally, SCFAs could lower IgA synthesis and improve hyperactive immune responses.^60^ Therefore, COS and COSF ameliorated IgAN progression by targeting specific microbial community associated with the intestinal barrier and mucosal immunity. COS intervention alleviated IgAN-induced B cell overactivation and modulated IgA secretion, increasing the expression of intestinal tight junction proteins ZO-1 and Occludin. These findings underscored the importance of maintaining a balance of specific microbial community for intestinal barrier and mucosal immunity in influencing IgAN progression.

In summary, IgAN led to structural and diversity changes in the gut microbiota. Alterations in specific microbial community associated with the intestinal barrier and mucosal immunity contributed to IgAN development. Targeted modulation of the specific microbial community improved the intestinal permeability (ZO-1 and Occludin) and rectified intestinal B cell immune imbalance (APRIL/BAFF and IgA levels) in IgAN and HMA-IgAN models, alleviating renal function damage. Therefore, specific microbial community related to the intestinal barrier and mucosal immunity are viable targets for regulating IgAN. However, there are some limitations to this study. While it elucidated the role of changes in specific microbiota in the occurrence and development of IgAN, it does not explore the molecular mechanisms leading to gut microbiota dysbiosis. Future research will focus on the molecular processes underpinning alterations in microbiota composition resulting from immune system dysfunction and host-microbiota interactions. Additionally, given the varying degrees of kidney damage at different stages of IgAN, further investigation is necessary into the dynamic changes in gut microbiota and their associated molecular mechanisms. Finally, the specific microbial community targeted by COS are also involved in multiple metabolic pathways, the interaction between the microbiota and the intestinal barrier and renal function needs to be further explored and confirmed in clinical practice.

## Materials and methods

### COS intervention in IgAN animal model experiments

All procedures involving experimental animals in this study were conducted in accordance with the protocol approved by the Animal Research Committee of East China University of Science and Technology (ECUST) (Ethical No. ECUST-2023-051), in compliance with the “Guide for the Care and Use of Laboratory Animals”. Specific pathogen-free (SPF) male Balb/c mice (5 weeks old) were purchased from Shanghai JieSiJie Laboratory Animal Co., Ltd. (Shanghai, China) (License No. SCxK 2023–0004). Mice were housed in a clean room with a temperature of 21 ± 2°C and a 12-hour light/12-hour dark cycle. Following a one-week acclimatization period, the mice were randomly assigned to five groups (*n* = 6): Control group (PBS), IgAN model group, COS-H group (350 mg/kg BW), COS-L group (50 mg/kg BW), and Positive (Prednisolone) group (5 mg/kg BW). COS used in this study (purity >95%, deacetylation > 98%, MW = 663.29 Da) was prepared according to the method of Li et al.^61^ (Figure S4, S5). Prednisolone was purchased from MedChemExpress (MCE, Shanghai, China) with a purity ≥ 99%. The mice were provided with free access to water and a standard diet. The IgAN animal model was established using the combination method of bovine serum albumin (BSA), LPS, and CCl_4_.^62,63^ Specifically, except for the control group, the remaining mice were gavaged with BSA (800 mg/kg BW) every other day for 8 weeks, received subcutaneous injections of castor oil (0.5 mL) and CCl_4_ (0.1 mL) once a week for 9 weeks, and were administered intravenous injections of 0.05 mg LPS in the 6th and 8th weeks. Mice in the control group were treated with an equivalent volume of PBS at corresponding times. All interventions were carried out after the modeling period, with daily gavage continuing for 6 weeks. At the end of the experiment, fresh feces were collected from the mice for gut microbiota diversity analysis. Mice were euthanized by cervical dislocation, and samples, including blood, intestinal tissue, and kidney tissue were collected for further analysis (Figure 1a).

### Subject sample collection and processing

This study recruited six patients with progressive IgAN and six healthy control (HC) volunteers (aged 20–60 years) (Table S1). All participants completed health questionnaires and signed consent forms. All experiments involving human subjects adhered to the Declaration of Helsinki. All experiments were conducted in accordance with the ethical committee of Minhang Hospital, Fudan University (Ethical No. 2021–056-01K). IgAN patients were diagnosed by renal biopsy from Minhang Hospital, Fudan University in Shanghai, China. Healthy volunteers were recruited from the medical examination center of the hospital. IgAN patients had not used immunosuppressants, corticosteroids, or antibiotics within 3 months before sample collection, and healthy control volunteers had no gastrointestinal diseases and had not used antibiotics or prebiotic products within 3 months. Fresh fecal samples from each volunteer were collected in sterile anaerobic tubes and promptly processed into fecal microbial suspensions. Within an anaerobic workstation (Thermo Fisher Scientific, Inc.), fecal samples from the IgAN and HC groups were mixed equally, respectively. Then prepared into a 50% (w/v) fecal suspension using the sterile PBS solution containing 20% sterile glycerol and thoroughly vortex-mixed. Fecal suspensions were filtered using double-layered gauze to remove food residues and then centrifuged at 600 × *g* at 4°C for 10 minutes to discard the supernatant and obtain the microbial precipitate. The precipitated organisms were resuspended in sterile PBS containing 20% sterile glycerol to get the fecal microbial suspension (OD_600_ = 1). The fecal microbial suspension was aliquoted for further use.

### Fecal microbial transplantation of IgAN patients and construction of an HMA mice model

To investigate the role of gut microbiota dysbiosis in the pathogenesis of IgAN, this study employed a HMA mice model, in accordance with the experimental protocol (Figure 1b). SPF male Balb/c mice (5 weeks of age) were purchased from Shanghai JieSiJie Laboratory Animal Co., Ltd. (Shanghai, China) (License No. SCxK 2023–0004). The mice were kept in a SPF-class housing of laboratory with a temperature of 21 ± 2°C and a 12-hour light/dark cycle. After a one-week acclimatization period, the mice were randomly divided, with one group designated as the IgAN model group (*n* = 6), and the remaining mice subjected to antibiotic treatment to establish a pseudo-germ-free model. The antibiotic treatment groups were administered a cocktail of broad-spectrum antibiotics daily via gavage (Shanghai Yuanye Bio-Technology Co., Ltd., Shanghai, China), consisting of vancomycin (100 mg/kg BW), neomycin sulfate (200 mg/kg BW), metronidazole (200 mg/kg BW), and ampicillin (200 mg/kg BW) for 3 weeks to establish a pseudo-germ-free mice model.^64,65^ Subsequently, the pseudo-germ-free mice were randomly divided into four groups (*n* = 6): HMA-HC group, HMA-IgAN group, COS group (350 mg/kg BW), and COSF group (350 mg/kg BW). Mice were gavaged every other day with 50 mg/0.2 mL of fecal suspension for 6 weeks. All interventions were conducted after the modeling period, with daily gavage continuing for 6 weeks. At the end of the experiment, fresh feces were collected for microbial diversity analysis. Mice were euthanized by cervical dislocation, and samples, including blood, intestinal tissue, and kidney tissue were collected for further analysis.

### Renal function assessment

Renal function indicators in serum and urine samples from each group were analyzed using commercial kits purchased from Nanjing Jiancheng Bioengineering Institute (Nanjing, China). Creatinine levels were measured using the Creatinine (Cr) Assay Kit (sarcosine oxidase, C011-2-1). Albumin levels were determined using the Albumin Assay Kit (Bromocresol Green, A028-2-1). Uric acid levels were measured using the Uric Acid (UA) Test Kit (enzyme colorimetry, C012-2-1). Urea nitrogen levels were determined using the Urea Assay Kit (urease, C013-2-1).

### Gut microbial diversity analysis

Fresh fecal samples from different treatment groups of mice were collected, and total DNA was extracted according to the manufacturer’s protocol of the Magnetic Soil and Stool DNA Kit (TIANGEN, China). High-throughput sequencing of the 16S full-length gene was performed on the PacBio sequencing platform with primers 27F (5′- AGAGTTTGATCMTGGCTCAG −3′) and 1492 R (5′- ACCTTGTTACGACTT −3′). USEARCH (version 10.0) was used to cluster sequences with similarity greater than 97% into Operational Taxonomic Units (OTUs), followed by taxonomic annotation (Naive Bayes classifier in QIIME2). The microbiome data analysis, including α-diversity, β-diversity, and inter-group difference analysis, was conducted on the BMK Cloud platform (www.biocloud.net.) (Beijing Biomarker Technologies Co., Ltd., China).

### Histological analysis

Fresh kidney tissues were fixed in 4% paraformaldehyde overnight, subsequently embedded in paraffin and sectioned (4 μm), and then the sections were stained with PAS and imaged using an upright optical microscope (Nikon Eclipse E100, Japan). The glomerular structure and inflammatory infiltration were then evaluated.

The fixed colon was embedded in paraffin and sectioned (4 μm), and then stained with hematoxylin and eosin (H&E) for nuclei and cytoplasm, respectively. After dehydration and coverslipping, images were captured under a microscope (Nikon Eclipse E100, Japan). Then the intestinal mucosa structure and inflammatory infiltration were assessed.

### Immunofluorescence and immunohistochemical analysis

Fresh kidney or colon tissues were fixed in 4% paraformaldehyde overnight, embedded in paraffin, and sectioned (4 μm). The sections were deparaffinized to water for antigen activity restoration. Sections were blocked with 3% BSA solution for 30 minutes, then incubated overnight at 4°C with primary antibodies Rabbit Anti-Mouse IgA (bs-0774 R), ZO-1 (Servicebio, GB111981), Occludin (Servicebio, GB111401), followed by incubation with fluorescently labeled secondary antibody 488-goat anti-rabbit (Servicebio, GB25303) in darkness at room temperature for 50 minutes. Autofluorescence was quenched before counterstaining nuclei with DAPI and mounting the sections. Imaging and analysis were performed using a Nikon Eclipse C1 upright fluorescence microscope equipped with a Nikon DS-U3 imaging system (Nikon, Japan).

Colon tissues fixed in 4% paraformaldehyde were embedded in paraffin and sectioned (4 μm). The sections were deparaffinized to water for antigen recovery. Sections were incubated at room temperature in the dark with 3% hydrogen peroxide solution for 25 minutes to block endogenous peroxidase activity. After blocking with 3% BSA, sections were incubated overnight at 4°C with rabbit anti-mouse IgA antibody (bs-0774 R, Bioss), rabbit polyclonal anti-C3 (21337–1-AP, Proteintech), and goat anti-mouse IgG H&L (FITC) (HY-P80950, MedChemExpress), then incubated with Goat Anti-Rabbit IgG H&L (FITC) (HY-P80951, MedChemExpress) at room temperature for 50 minutes. After DAB staining and nuclear counterstaining, imaging and analysis were performed under a Nikon E100 microscope (Nikon, Japan).

### Quantitative real-time PCR (qPCR) analysis

Total RNA from intestinal tissues of mice from different groups was extracted according to the manufacturer’s instructions of the FastPure Cell/Tissue Total RNA Isolation Kit V2. The extracted total RNA was then reverse-transcribed following the manufacturer’s instructions for HiScript II Q RT SuperMix for qPCR (+gDNA wiper) to be used in qPCR. The qPCR analysis was then conducted using AceQ^Ⓡ^ qPCR SYBR Green Master Mix. The mRNA expression levels of each gene were normalized to GAPDH and calculated using the ΔΔCt method. The primers used for the genes were listed in Table S2.

### Enzyme-linked immunosorbent assay (ELISA) and colorimetric assay kit

Whole blood from mice in each group was collected, left at room temperature for 2 hours, and then centrifuged at 4°C, 3000 × *g* for 15 minutes to collect the supernatant. BAFF, APRIL, LPS, and DAO levels were determined using ELISA kits (Shanghai Enzyme-linked Biotechnology Co., Ltd.), and the absorbance of the developed color was measured at OD_450_. D-LA was determined using a colorimetric assay kit (Elabscience), with the absorbance of the developed color measured at OD_530_. Cecal tissue from each group of mice was collected, homogenized at 4°C, and then centrifuged at 1000 × *g* for 15 minutes to collect the supernatant for ELISA detection of IgA. Protein concentrations were quantified using the Enhanced BCA Protein Assay Kit (Shanghai Beyotime Biotechnology Co., Ltd.). All assays were conducted according to the manufacturer’s instructions.

### Correlation analysis of the key gut microbiota and phenotypic indicators of IgAN

The RDA analyses and Spearman’s correlation analysis were performed using normalized OTU abundance (including *Clostridium leptum*, *unclassified Roseburia*, *Streptococcus acidominimus*, etc.) and phenotype indicators of IgAN (including Scr, ACR, IgA, etc.) (6 samples per group). RDA analyses were performed using the Envfit function of the Vegan package in R (version 3.6.3) (http://cran.r-project.org/).^66,67^ Species with mean relative abundance greater than 1% were considered dominant species and subjected to Spearman’s correlation analysis with the phenotypic indicators (6 samples per group). The *p* values were adjusted using the Benjamini-Hochberg method for controlling the FDR.^68^ The interaction network was visualized using Cytoscape (version 3.8.0).

### Statistical analysis

All measurements were presented as mean ± standard deviation (SD). The t-test was used to compare statistical differences between the two groups, while one-way ANOVA with Tukey’s post hoc test was used for differences among multiple groups. Image J (version 1.8.0) was used to calculate the staining area in three independent immunofluorescence experiments. * Represented *p* < 0.05, ** represented *p* < 0.01, and *** represented *p* < 0.001, ns indicated no significant difference.

## Supplementary Material

Supplementary material.docx

## Data Availability

The data that support the findings of this study are available in https://doi.org/10.6084/m9.figshare.25572453, and within the article and its supplementary materials.
