# Supplementary material for: Targeted modulation of intestinal barrier and mucosal immune-related microbiota attenuates IgA nephropathy progression
Source: Gut Microbes. 2025 Jan 28;17(1):2458184. doi: 10.1080/19490976.2025.2458184 (PMC11776482; doi:10.1080/19490976.2025.2458184)
Supplement: Supplementary material.docx [file KGMI_A_2458184_SM0872.docx]

**Supplementary Information**

**Supplementary Results**

**Analysis of gut microbiota in pre-transplant IgAN patients and healthy controls**

The results indicated that the α diversity indices (Chao1 and Shannon) of the gut microbiota in IgAN patients were significantly lower than those in healthy controls (*p* < 0.05) (Figure S1A). Additionally, β diversity analysis (NMDS, PCA, PCoA and hierarchical clustering analysis) revealed differences in microbiota composition between IgAN patients and HCs (Figure S1B-E). The IgAN group was distinguishable from the HC group, with hierarchical clustering analysis providing an intuitive display of these differences. Among the top 10 genera, *Shigella*, *Bacteroides*, and *Streptococcus* were more abundant in the IgAN group, whereas *Blautia*, *Bifidobacterium*, and *Coprococcus* were more abundant in the HC group.

LEfSe analysis further highlighted significant differences in microbiota across various taxonomic levels between the two groups (Figure S1F, G). At the phylum level, *Proteobacteria* was significantly more abundant in the IgAN group. At the genus level, the IgAN group had significantly higher abundances of *Shigella*, *[Ruminococcus]*, *Erysipelotrichaceae_Clostridium*, *Eggerthella* and *Halomonas*, and significantly lower abundances of *Bifidobacterium*, *Alistipes*, *Oscillospira*, *Dialister*, *Roseburia* and *Anaerostipes* compared to the HC group. Further comparison at the species level (Figure S1H) showed that *unclassified Shigella* was significantly elevated in the IgAN group, while *Bifidobacterium_pseudolongum*, *Parabacteroides_gordonii*, *unclassified_Clostridiales*, *unclassified_Clostridium*, *unclassified_Lachnospiraceae*, *unclassified_Roseburia*, *unclassified_Ruminococcaceae*, *unclassified_Odoribacter*, *unclassified_Oscillospira* were significantly less abundant.


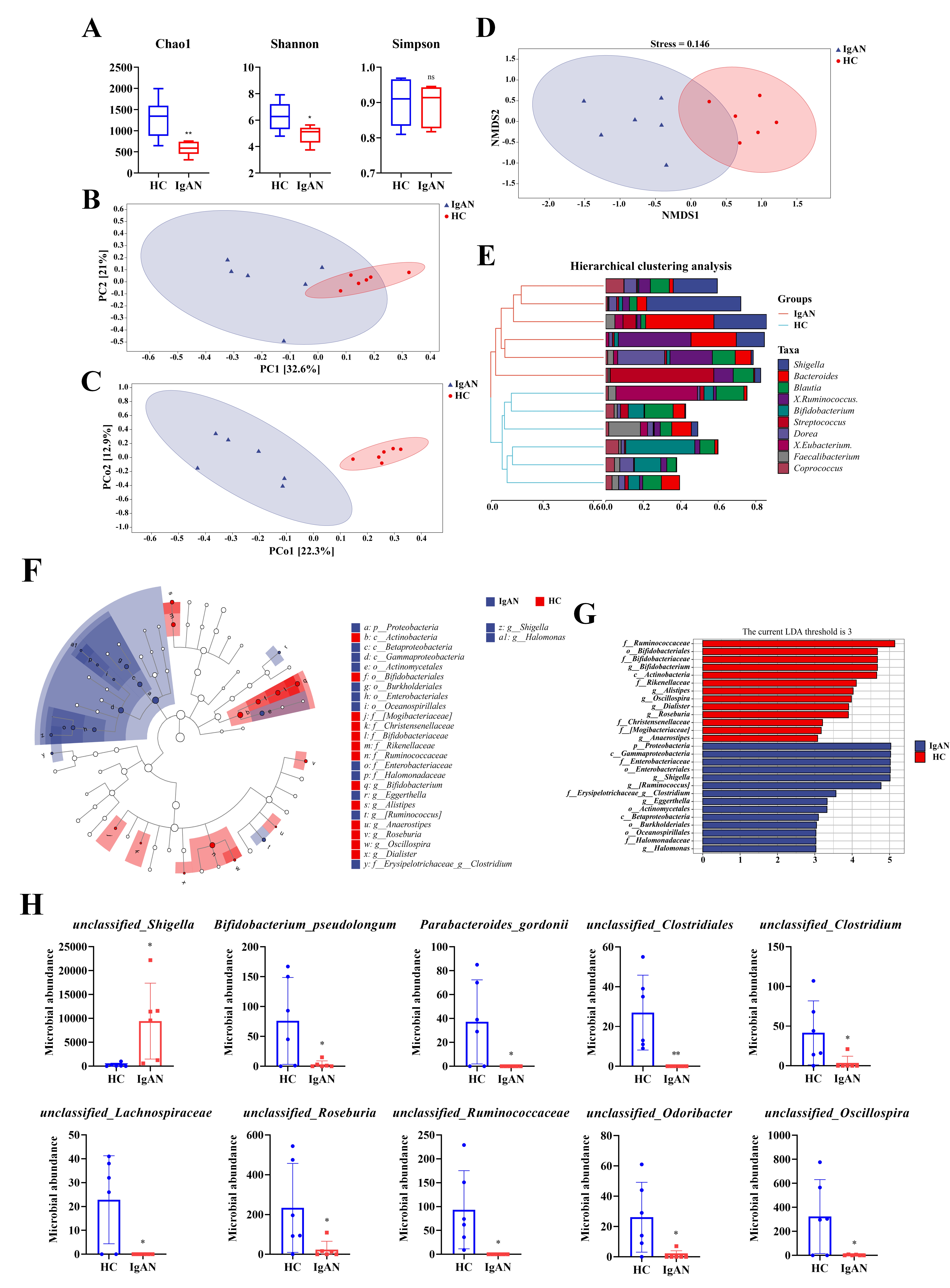


**Figure S1** Analysis of gut microbiota differences between IgAN patients and healthy control volunteers used for HMA animal construction (n = 6). **A** α-diversity analysis. **B** PCA analysis. **C** PCoA analysis. **D** NMDS analysis. E Hierarchical clustering analysis. **F-G** LEfSe analysis. **H** Comparison of species-level differential taxa. * Represented *p* < 0.05, and ** represented *p* < 0.01.

**Serum and fecal metabolomic analysis of pre-transplant IgAN patients and healthy controls**

The overall clustering analysis revealed the aggregation of the serum and fecal metabolome of IgAN patients and HC groups (Figure S2-3). Both PCA and PLS-DA analyses indicated significant shifts in the serum and fecal metabolomes of IgAN patients compared to the control group, particularly in the serum metabolome. Further analysis of the serum differential metabolites in IgAN patients identified significant elevations in metabolites related to inflammation and immune response, such as L-Glutamate, 5-HETE, and 14-HDoHE. Metabolites associated with energy metabolism disorders, including nicotinate D-ribonucleoside, cis-2-methylaconitate, and fumaric acid were also significantly elevated. Additionally, metabolites related to signal transduction, such as N-acetylneuraminic acid, sphingosine-1-phosphate, 2-arachidonoyl glycerol, and disialosyl galactosyl globoside, were significantly increased. The level of the urinary toxin phenol sulfate was also notably elevated. In contrast, IgAN patients exhibited significantly lower levels of several bile acids, including dehydrocholic acid, sulfolithocholylglycine, lithocholic acid glycine conjugate, and polyunsaturated fatty acids.

In the feces of IgAN patients, the levels of vitamins and the derivatives, including nicotinamide and vitamin B6, were significantly elevated. The increased levels of lipid metabolites, particularly those involving cholesterol and bile acid derivatives (such as N-arachidonoyl GABA, dihydrocholesterol, cholesterol glucuronide, and deoxycholic acid glycine conjugate), reflected enhanced lipid absorption and metabolism in the intestine. This may be a compensatory response to kidney disease, where the intestine attempts to counteract the loss of kidney function by increasing lipid metabolism. The level of urinary toxin 4-ethylphenyl sulfate was significantly higher in the IgAN group, suggesting that the gut microbiota of IgAN patients was dysfunctional. Furthermore, analysis of lipid-related metabolites in the serum and fecal metabolome revealed significant lipid metabolic disorders in IgAN patients, particularly in phospholipid metabolism, which may indicate substantial changes in cell membrane structure and signaling pathways.

KEGG analysis of the differential metabolites in the serum and feces of the two groups revealed that IgAN patients exhibited significant alterations in aromatic amino acid metabolism, lipid signaling, and energy-related metabolism. In summary, IgAN patients not only experience disruptions in the diversity and structure of their gut microbiota but also show changes in their metabolic characteristics.


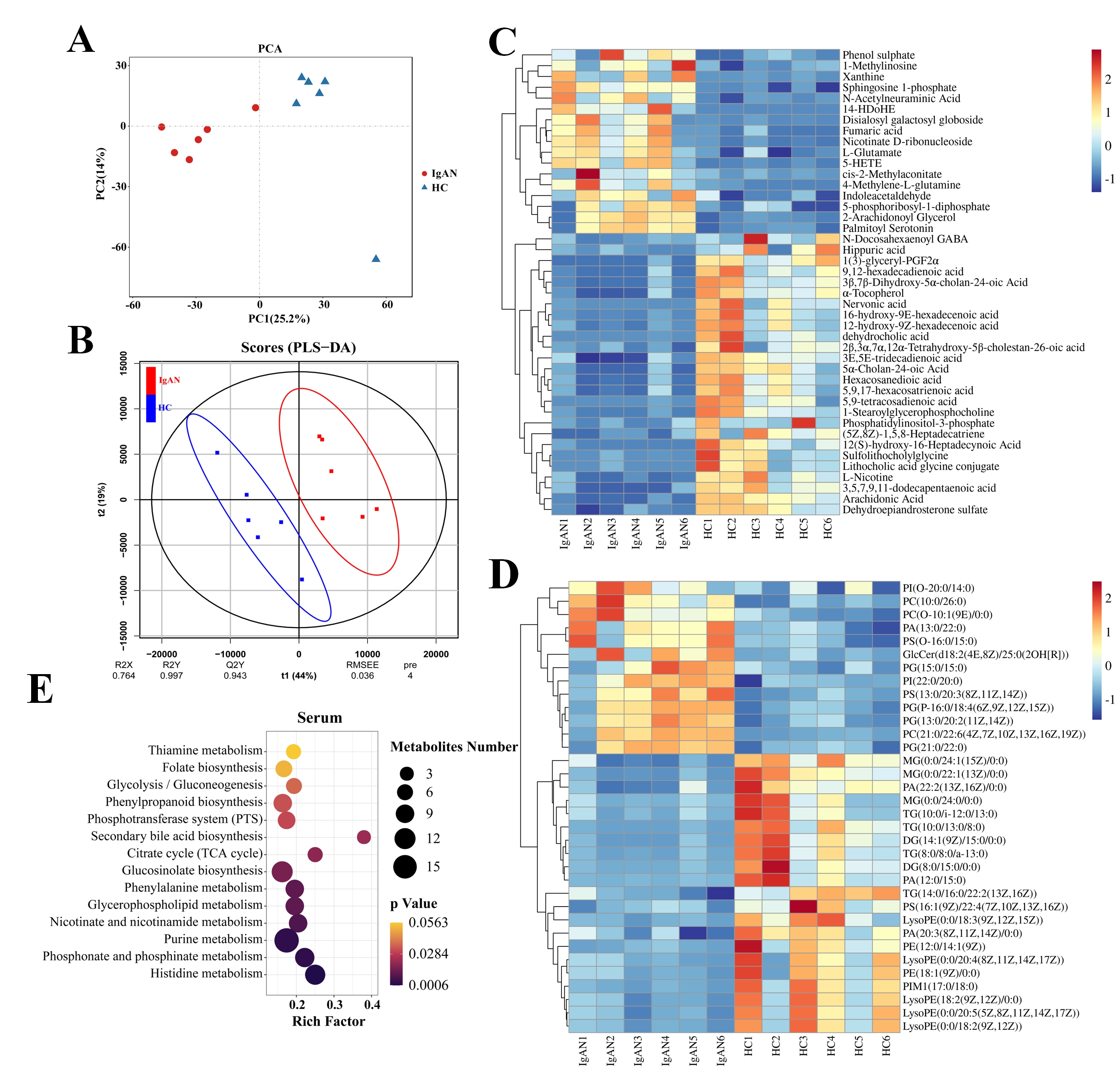


**Figure S2** Analysis of serum metabolomic differences between IgAN patients and healthy control volunteers used for HMA animal construction (n = 6). **A** PCA analysis. **B** PLS-DA analysis. **C-D** Screening of serum differential metabolites. **E** KEGG pathway analysis of serum differential metabolite.


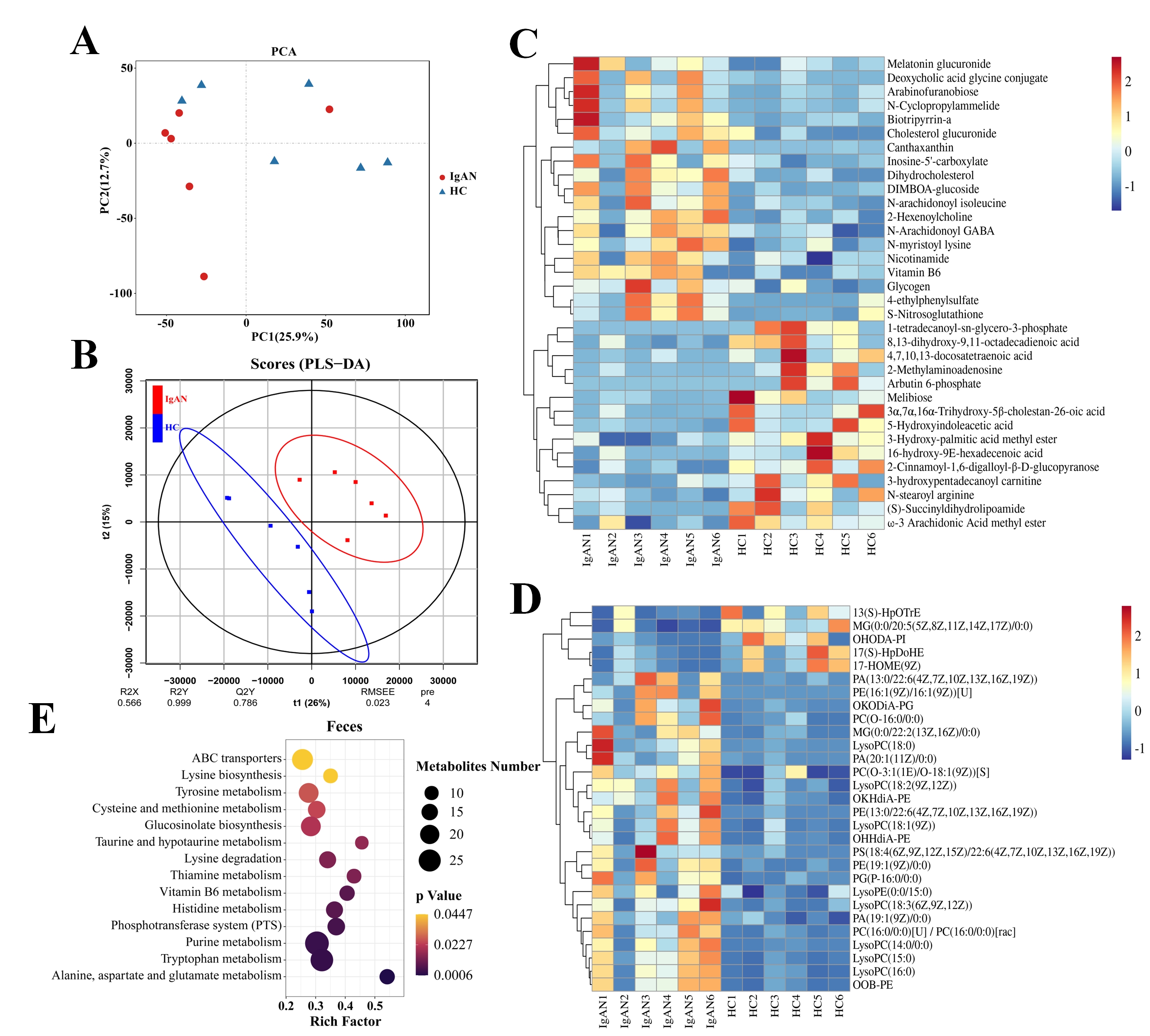


**Figure S3** Analysis of fecal metabolomic differences between IgAN patients and healthy control volunteers used for HMA animal construction (n = 6). **A** PCA analysis. **B** PLS-DA analysis. **C-D** Screening of fecal differential metabolites. **E** KEGG pathway analysis of fecal differential metabolite.

Figure. S4


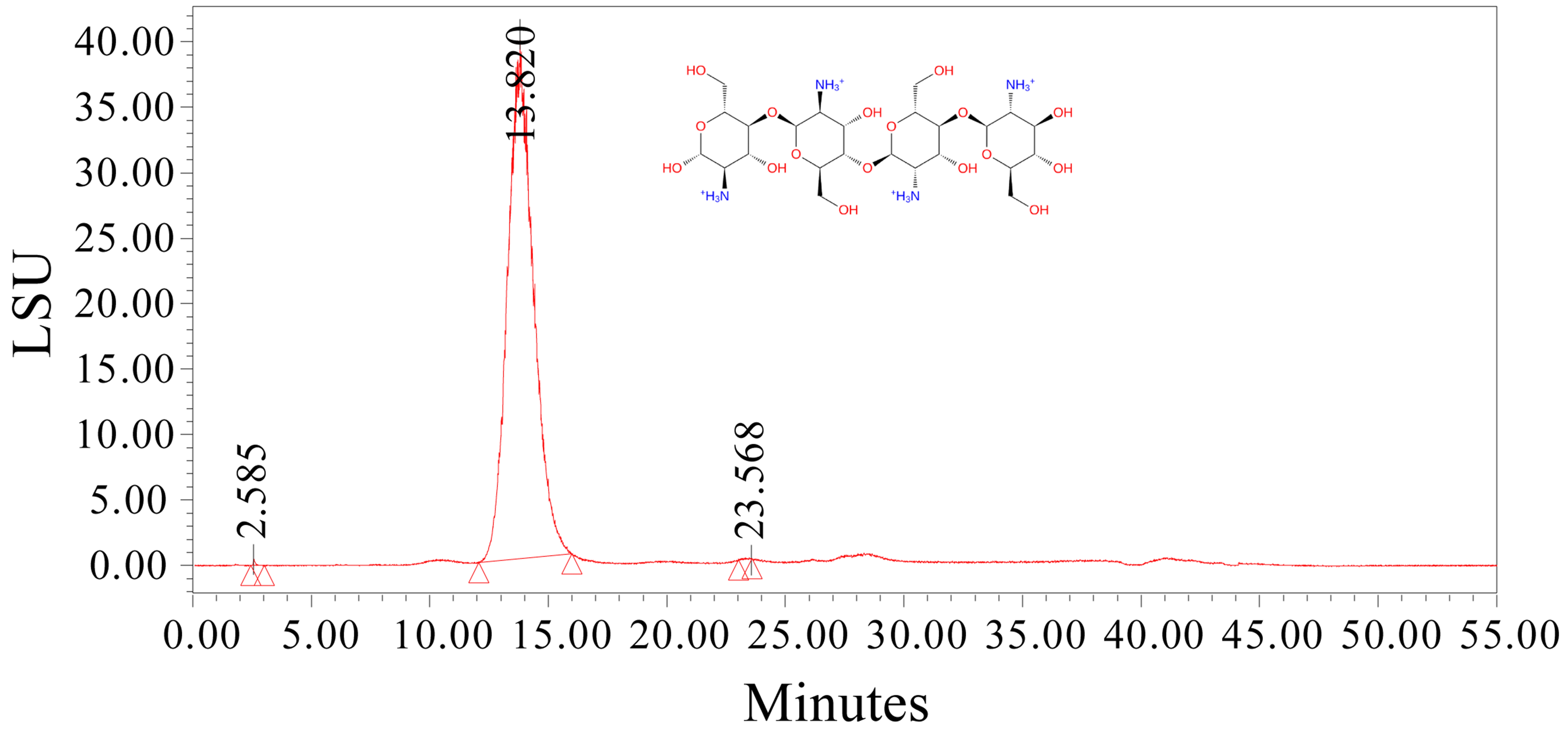


**Figure. S4. HPLC-ELSD determination of COS4.** The purity of COS4 was greater than 98% compared to the standard and the retention time of COS4 was 13.820 min.

Figure. S5


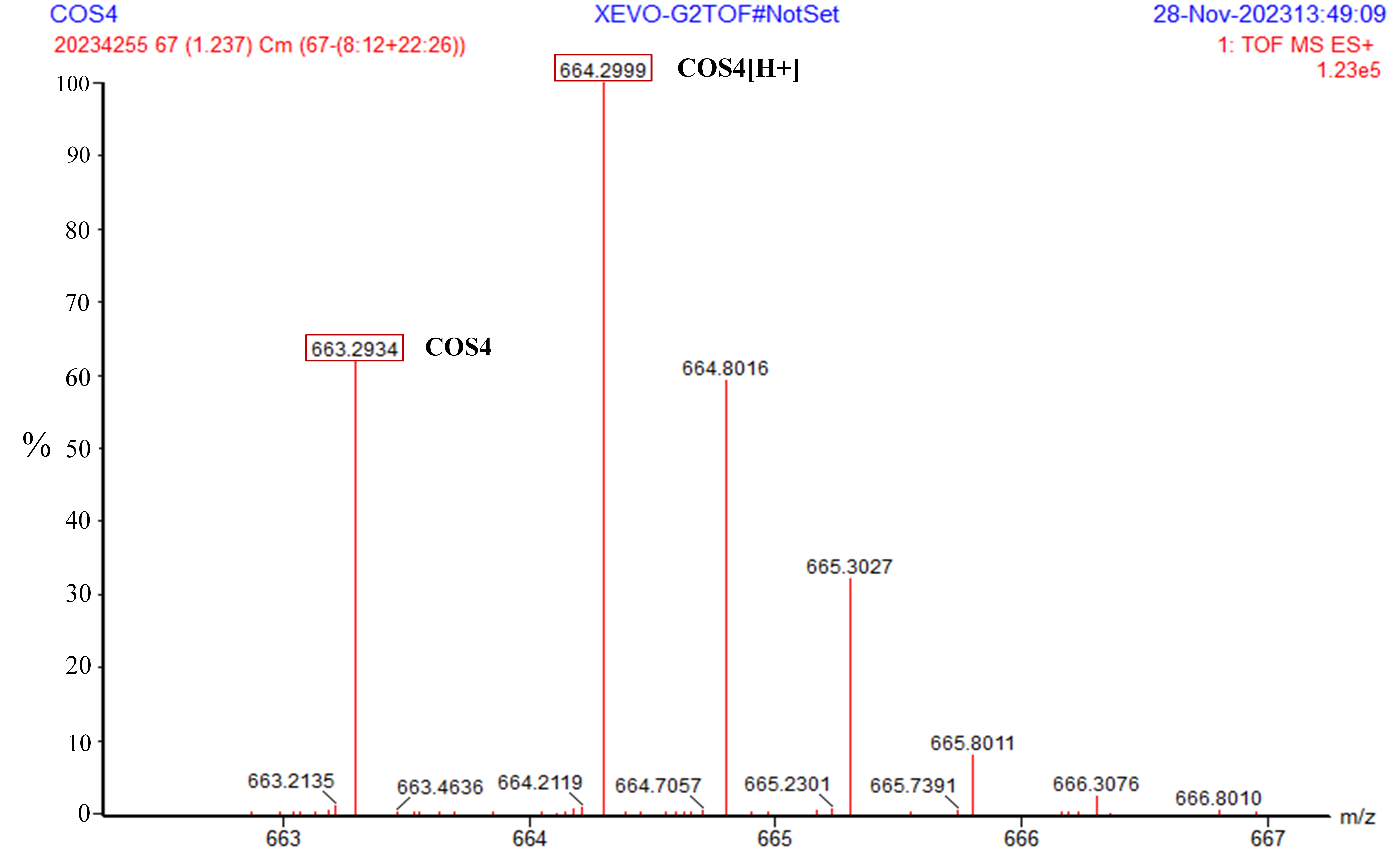


**Figure. S5. ESI-high-resolution time-of-flight mass spectrometry determination of COS4.** The molecular weight of COS4 is 663.2934.

**Table S1. The baseline indices of 12 volunteers.**

| Baseline Indices | HC1 | HC2 | HC3 | HC4 | HC5 | HC6 | IgAN1 | IgAN2 | IgAN3 | IgAN4 | IgAN5 | IgAN6 | Sig. |  |
| --- | --- | --- | --- | --- | --- | --- | --- | --- | --- | --- | --- | --- | --- | --- |
| sex | 0 | 0 | 0 | 1 | 1 | 1 | 0 | 0 | 0 | 1 | 1 | 1 | ns |  |
| age | 30 | 37 | 30 | 25 | 30 | 30 | 28 | 36 | 30 | 42 | 34 | 29 | ns |  |
| BMI | 22.0 | 20.8 | 29.4 | 25.0 | 20.4 | 22.1 | 24.1 | 27.3 | 31.8 | 23.9 | 27.0 | 27.2 | ns |  |
| Scr  (umol/L) | 65.0 | 67.0 | 80.0 | 55.0 | 43.0 | 45.0 | 98.0 | 155.0 | 72.0 | 82.0 | 86.0 | 129.0 | * |  |
|  |  |  |  |  |  |  |  |  |  |  |  |  |  |  |
| eGFR | 128.5 | 122.6 | 113.4 | 132.9 | 140.0 | 129.1 | 67.7 | 37.0 | 97.8 | 101.0 | 101.5 | 64.1 | ** |  |
| Cys-C  (mg/L) | 0.4 | 0.7 | 0.6 | 0.6 | 0.5 | 0.5 | 1.1 | 1.7 | 1.1 | 0.9 | 1.1 | 1.5 | *** |  |
|  |  |  |  |  |  |  |  |  |  |  |  |  |  |  |
| BUN  (mmol/L) | 4.4 | 4.4 | 4.1 | 3.1 | 2.8 | 3.0 | 5.3 | 9.0 | 4.5 | 4.6 | 5.2 | 6.3 | * |  |
|  |  |  |  |  |  |  |  |  |  |  |  |  |  |  |
| HBA1C | 4.8 | 4.3 | 4.2 | 4.5 | 4.0 | 4.3 | 6.7 | 6.8 | 7.0 | 6.8 | 6.5 | 6.5 | **** |  |
| 24h-upro（mg） | 0.1 | 0.1 | 0.1 | 0.1 | 0.1 | 0.1 | 1.9 | 3.9 | 1.4 | 9.9 | 1.5 | 1.1 | * |  |
|  |  |  |  |  |  |  |  |  |  |  |  |  |  |  |

Abbreviations: BMI, body mass index. Scr, serum creatinine. eGFR, estimated glomerular filtration rate. Cys-C, Cystatin C. BUN, blood urea nitrogen. HBA1C, hemoglobin A1C. 24h-upro, 24h urine proteinuria.

Table S2. Primer pairs for qPCR.

| **Gene** | **Primer** | **Sequence** |
| --- | --- | --- |
| *GAPDH* | Forward | CCACTCACGGCAAATTCAAC |
|  | Reversed | GTAGACTCCACGACATACTCA |
| *ZO-1* | Forward | ACCCGAAACTGATGCTGTGGATAG |
|  | Reversed | AAATGGCCGGGCAGAACTTGTGTA |
| *Occludin* | Forward | GGTGAATGGGTCACCGAGGG |
|  | Reversed | AGCAAAATGTCCAGGCTCCC |

Table S3. Comparison of trends in gut microbiota at the species level between groups with significant differences.

| **Groups** | **Species** | **Changes** | **Groups** | **Species** | **Changes** |
| --- | --- | --- | --- | --- | --- |
| IgAN *vs* Control | *Alistipes massiliensis* | ⭡ | COS *vs* IgAN | *Afistipes massilieusis* | ⭣ |
|  | *Shigella sonnei* | ⭡ |  | *Shigella sonnei* | ⭣ |
|  | *Streptococcus danieliae* | ⭡ |  | *Streptococcus danieliae* | ⭣ |
|  | *Streptococcus thoraltensis* | ⭡ |  | *Streptococcus thoraltensis* | ⭣ |
|  | *Desulfovibrio fairfieldensis* | ⭡ |  | *Bacteroides acidifaciens* | ⭣ |
|  | *Alistipes putredinis* | ⭡ |  | *Adlercreutzia muris* | ⭣ |
|  | *Phocaeieola vulgatus* | ⭣ |  | *Acutalibacter sp.* | ⭡ |
|  | *Clostridium sp. SN17* | ⭣ |  | *unclassified Lachnospiraceae NK4Al36 group* | ⭡ |
|  | *Ruminococcacene UCG-005* | ⭣ |  | *Clostridium sp. Culture-41* | ⭡ |
|  | *unclassified Ruminococcaceae* | ⭣ |  | *unclassified Lachnospiraceae* | ⭡ |
|  | *unclassified Lachnocostridium* | ⭣ |  | *unclassified Odoribacter* | ⭡ |
|  | *unclassified Prevoellaceae UCG-001* | ⭣ |  | *unclassified Oscillospirales* | ⭡ |
| Humanized IgAN *vs* Humanized Control | *Bacteroides sp. SLC1-38* | ⭡ | COSF *vs* Humanized IgAN | *Bacteroides sp. SLC1-38* | ⭣ |
|  | *Parabacteroides gordonii* | ⭡ |  | *Parabacteroides gordonii* | ⭣ |
|  | *unclassified Anaerofustis* | ⭡ |  | *Paresntterela ereremenfihominis* | ⭣ |
|  | *unclassified Erysipelatoclostridium* | ⭡ |  | *unclassified Erysipelatoclostridium* | ⭣ |
|  | *Bifdobacteriun pseudolongum* | ⭣ |  | *Clostridium leptum* | ⭡ |
|  | *Clostridium leptum* | ⭣ |  | *unclassified Clostridia vadinBB60 group* | ⭡ |
|  | *Acutalibacter muris* | ⭣ |  | *unclassified Oscillospiraceae* | ⭡ |
|  | *unclassified Oscillibacter* | ⭣ |  | *unclassified Roseburia* | ⭡ |
|  | *unclassified Roseburia* | ⭣ |  | *unclassified Rikenela* | ⭡ |
|  | *unclassified Ruminococcaceae* | ⭣ |  | *unclassified Oscillibacter* | ⭡ |
|  | *unclassified Odoribacter* | ⭣ |  | *unclassified UCG 010* | ⭡ |
